# Infant hippocampal and amygdala volume in an observational follow-up from a randomized clinical trial for maternal prenatal depression

**DOI:** 10.1038/s41467-026-78276-4

**Published:** 2026-10-16

**Authors:** Catherine H. Demers, Benjamin L. Hankin, LillyBelle K. Deer, Robert J. Gallop, Mercedes Hoeflich Haase, M. Camille Hoffman, Martin A. Styner, Nancy K. Grote, Elysia Poggi Davis

**Affiliations:** 1 University of Colorado Anschutz Medical Campus, Department of Psychiatry, Aurora, CO USA; 2 University of Denver, Department of Psychology, Denver, CO USA; 3 University of Illinois at Urbana-Champaign, Department of Psychology, Champaign, IL USA; 4 University of Utah, Department of Psychology, Salt Lake City, UT USA; 5 West Chester University, Department of Mathematics, West Chester, PA USA; 6 University of North Carolina – Chapel Hill, Department of Psychiatry, Chapel Hill, NC USA; 7 University of Colorado Denver School of Medicine, Department of Obstetrics and Gynecology, Division of Maternal and Fetal Medicine, Aurora, CO USA; 8 University of North Carolina – Chapel Hill, Department of Computer Science, Chapel Hill, NC USA; 9 Department of Biomedical Engineering, University of Basel, Allschwil, Switzerland; 10 University of Washington, Department of Social Work, Seattle, WA USA; 11 University of California, Irvine, Department of Pediatrics, Irvine, CA USA

**Keywords:** Developmental biology, Human behaviour

## Abstract

Prenatal maternal depression may have intergenerational effects on offspring brain development, with potential socioemotional consequences. This observational follow-up study examines brain development and subsequent negative emotionality among 109 infants born to mothers participating in a randomized clinical trial (NCT03011801). In the prior trial, brief interpersonal psychotherapy significantly reduced prenatal depression symptoms and disorder as the published primary and secondary endpoints. Exploratory infant outcomes include hippocampal and amygdala volumes at 6 weeks and negative emotionality at 6 months. Here we show that infants of mothers randomized to interpersonal psychotherapy during pregnancy have lower right hippocampal volume than infants of mothers in the enhanced usual care group (*p* = .002, *d* = 0.62). No statistically significant group differences are observed in left hippocampal or amygdala volume. Smaller hippocampal volume predicts subsequent lower negative emotionality (r = 0.19, *p* = 0.045). In this study, a maternal prenatal depression intervention is associated with differences in infant brain development with implications for their subsequent emotional health.

## Abstract (web summary)

Depression during pregnancy may influence infant brain development and emotional health. In a follow-up of an RCT, infants whose mothers received depression treatment had smaller right hippocampal volumes, which predicted lower negative emotionality.

## Introduction

The remarkable transformation from a single-celled zygote to a fully developed human newborn is profoundly influenced by environmental factors, particularly during sensitive periods of fetal brain development. Research spanning decades has established a compelling link between prenatal maternal mental health and long-term offspring health outcomes, supporting the Fetal or Developmental Origins of Health and Disease Hypothesis^1^. However, existing human research has been limited by its reliance on correlational evidence. Experimental control through intervention is needed^2^. As an observational follow-up of a randomized clinical trial (RCT), the current study evaluates neurodevelopmental outcomes of infants whose mothers participated in an effective depression intervention during pregnancy^3^. This research identifies differences in brain structure of infants born to mothers randomized to the active intervention group relative to the enhanced usual care group, highlighting the intergenerational impact of prenatal mental health treatment.

Maternal depression is one of the most common prenatal complications, with 20-40% of individuals reporting elevated symptoms of depression during pregnancy^4,5^. Rates of depression are even higher among socioeconomically at-risk populations^6,7^. Longitudinal correlational studies show robust intergenerational associations between maternal depression and a range of adverse child developmental outcomes, including socioemotional and cognitive difficulties and a nearly 3-fold increased risk of psychopathology^8–12^. Experimental evidence in rodents^13^ and correlational studies in humans suggest that prenatal exposure to maternal depression, a form of early life adversity, has cascading effects on offspring brain development^14^, particularly within limbic regions such as the amygdala and the hippocampus (for reviews see refs. ^15–17^). The amygdala and hippocampus are two prime candidate regions for investigating the effects of prenatal depression exposure given their critical roles in affective processing, fear learning, and memory^18–21^, as well as established links with many psychiatric illnesses, including post-traumatic stress disorder and depression^22–25^. Both regions are highly susceptible to stress and contain high levels of glucocorticoid receptors, increasing their sensitivity to dysregulation of the hypothalamic–pituitary–adrenal (HPA) axis^26^. Cross-species evidence indicates these regions are vulnerable to adversity, including maternal depression, during the prenatal period. In humans, exposure to maternal prenatal depression and distress is associated with volumetric alterations in the amygdala and hippocampus in infancy^27–32^ as well as later childhood and adolescence^33–35^. The vulnerability of these regions is substantiated by experimental rodent and non-human primate research demonstrating that maternal prenatal stress impacts amygdala and hippocampal morphology^13,36^.

Emerging research assessing neurodevelopment provides evidence that early-life adversity may be linked to accelerated maturation or rapid growth during early life as an adaptive response^37^. Consistent with this hypothesis, correlational findings suggest that exposure to prenatal maternal depression leads to enlarged subcortical volumes^27^, including hippocampal and amygdala volume^28^ in infancy; however, see also^29,38–40^. While accelerated maturation may initially be adaptive in meeting the demands of the current environment^41^, neurodevelopmental changes may increase the lifetime risk of mental illness and, in fact, have been associated with neurodevelopmental and socioemotional outcomes such as increased negative emotionality and depressive symptoms^42,43^. The few existing studies linking maternal prenatal depression and limbic system development in early infancy are limited by their reliance on correlational designs. As called for by O’Connor et al. (2014)^2^, experimental control through intervention is needed to more directly test the links between prenatal depression and infant brain structure.

Results from our previously published findings have established that a brief, noninvasive psychosocial intervention (brief interpersonal psychotherapy (IPT)) rapidly and substantially reduces maternal prenatal depression before birth^50^. Specifically, the RCT examined depression symptoms across pregnancy among 234 individuals randomized to either brief IPT or enhanced usual care (EUC). Significant group differences were observed such that the IPT treatment group displayed a significantly greater decline in both symptoms and diagnosis rate compared to the EUC comparison group, the trial’s primary and secondary outcomes. Specifically, individuals in the IPT group showed a 47% decrease on the Symptom Checklist (SCL-20) and a 52.5% decrease on the Edinburgh Postnatal Depression Scale (EPDS) from baseline to the end of gestation; in contrast, those in the EUC condition showed a 13.0% (SCL-20) and 32.3% (EPDS) decrease in symptoms. Further, participants assigned to IPT showed significantly greater major depressive disorder (MDD) remission relative to EUC. MDD rate from baseline to the end of gestation decreased 5-fold from 36.5% to 6.1% for IPT participants, compared to a smaller decrease (37 to 26.1%) for EUC (odds ratio, 4.99; 95% CI, 2.08–11.97). Further, this intervention benefits birth outcomes, including longer gestation^44^. These findings provide a unique opportunity to examine whether offspring structural brain development differs among infants born to participants randomized to IPT versus EUC. Here, we evaluate hippocampal and amygdala volumes as exploratory offspring outcomes in an observational follow-up of this RCT.

The current study is an observational follow-up comparing infant brain structure in the offspring of mothers randomized to a prenatal depression treatment versus a comparison group. Infant hippocampal and amygdala volumes were evaluated with structural MRI shortly after birth. Secondary analyses examined possible mechanisms underlying any observed group differences. We tested whether change in depressive symptoms, as well as perceived stress and anxiety across pregnancy, were associated with infant brain volume. Additional analyses probed whether infant sex moderated intervention effects, given prior evidence for sex-specific vulnerability in neurodevelopmental outcomes following prenatal stress exposure^45,46^. Finally, we explored the developmental significance of early brain differences by testing whether neonatal hippocampal and amygdala volume predicted negative emotionality at 6 months, an early-emerging trait linked to increased risk for later mental health problems^47^. Negative emotionality was evaluated with standardized laboratory-based assessments.

Here, we show that infants born to mothers randomized to brief interpersonal psychotherapy during pregnancy have lower right hippocampal volume compared to infants whose mothers were randomized to enhanced usual care. Left hippocampal and bilateral amygdala volumes do not differ between groups. We further show that lower right hippocampal volume is associated with less infant negative emotionality at 6 months. These findings suggest that prenatal maternal depression intervention is associated with differences in early infant hippocampal development that relate to later negative emotionality. Study findings provide a framework for evaluating intergenerational effects of prenatal mental health interventions.

## Results

**Fig. 1:**
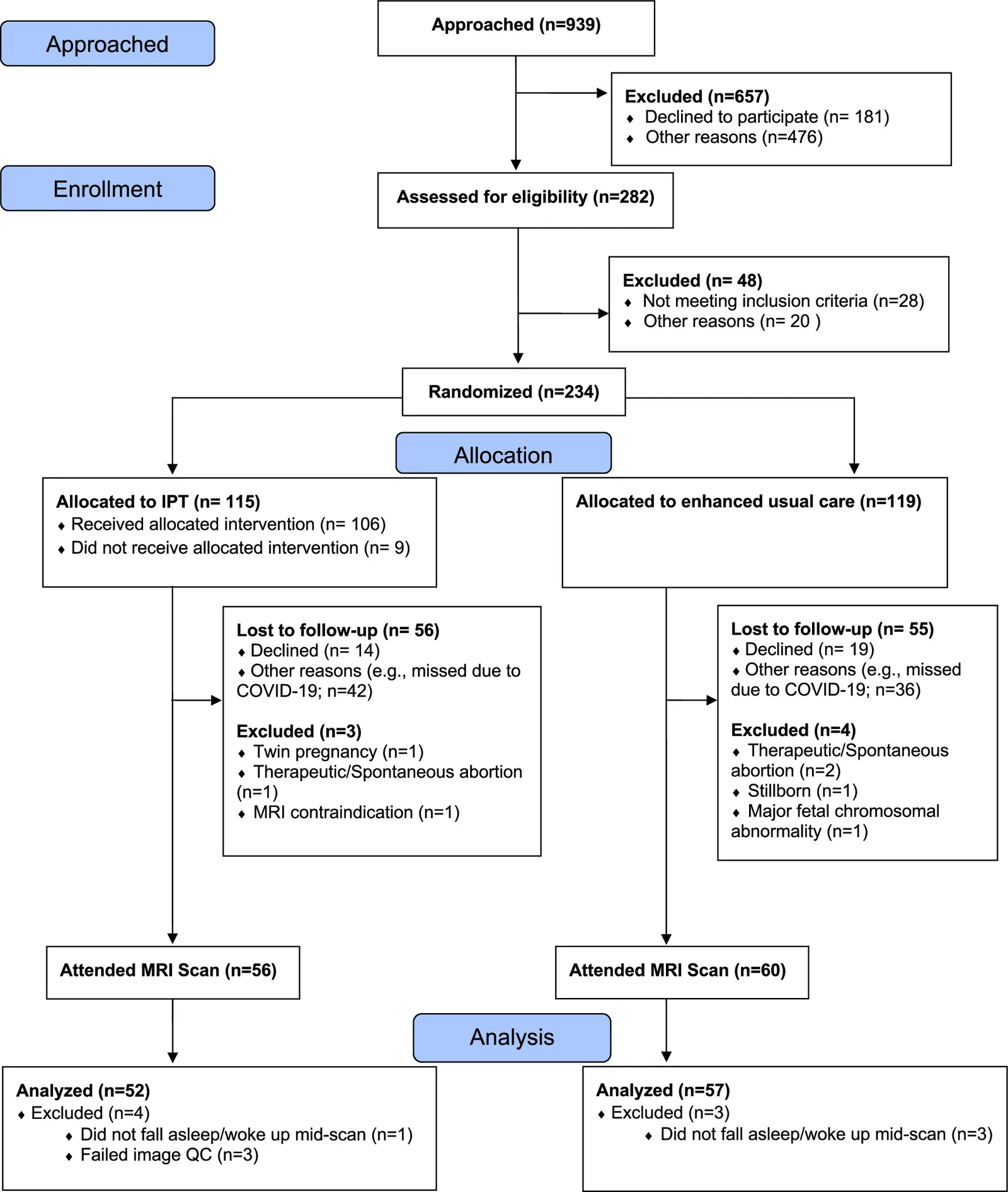
CONSORT diagram of the randomized clinical trial and observational infant follow-up. Participant flow through recruitment, randomization, infant MRI assessment, and final analytic sample.

**Fig. 2:**
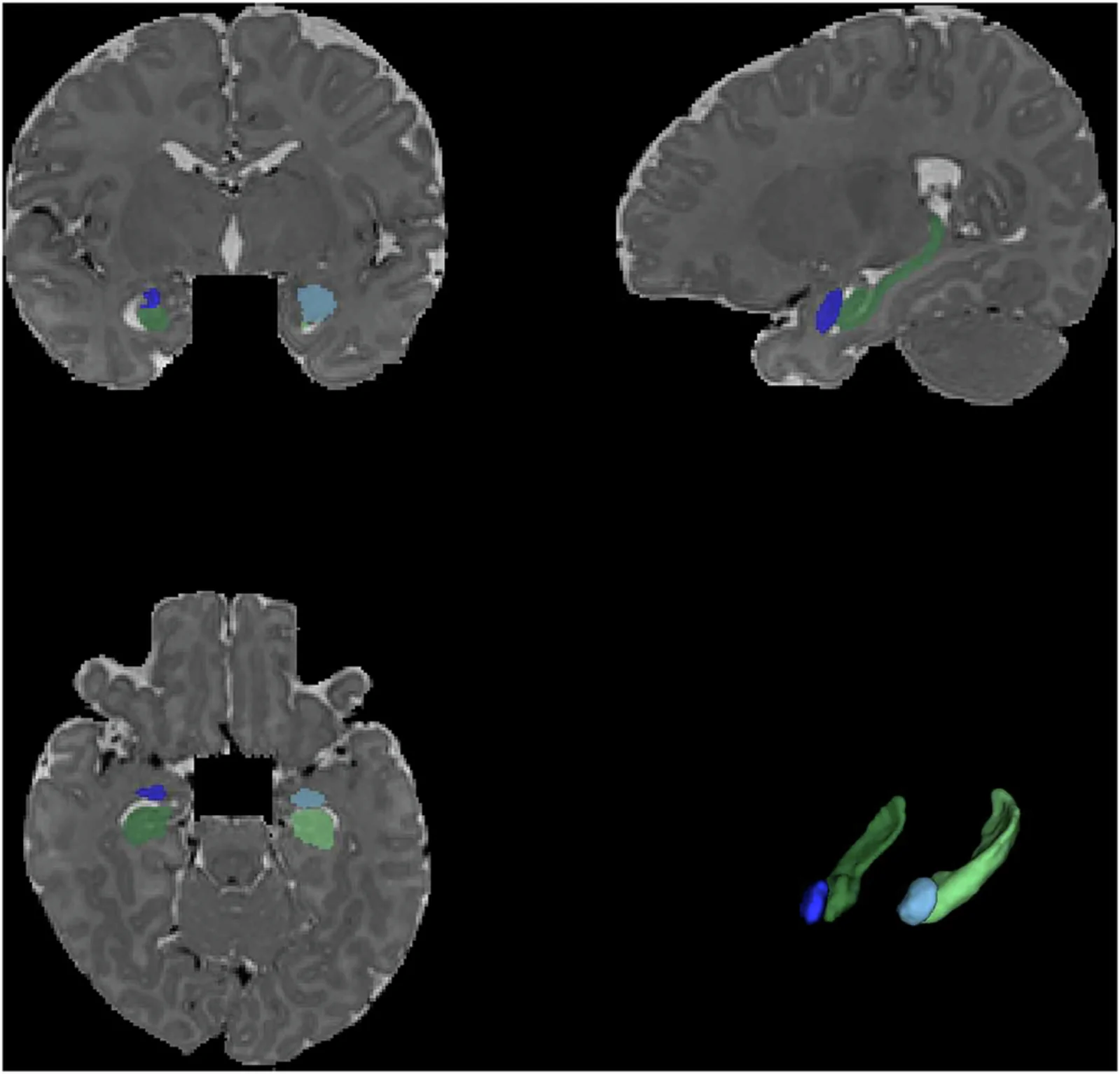
Bilateral hippocampal and amygdala regions of interest. 3D visualization of the amygdala and hippocampal segmentation on MRI T1 (T2) weighted scan. Green = hippocampus, Blue = amygdala. Brain volume metric is mm^3^.

Figure 1 provides the CONSORT diagram and illustrates allocation to intervention conditions and participant flow. Participants reported their race and ethnicity as follows: 18.3% Latine; 49.5% non-Hispanic/Latine white, 7.3% Black, 1.8% Asian, and 22.9% Multiracial and/or Multiethnic. Participants were 29.96 years of age and 18 weeks’ gestational age on average at recruitment. Participants were socioeconomically diverse, with 40.4% of participants living at or near the federal classification of poverty (less than 200% household income-to-needs ratio). Infants were scanned at 44.8 weeks postconceptional age on average (see Fig. 2 for amygdala and hippocampal regions of interest), and infant negative emotionality was assessed at 6 months of age. Participants reported on psychotropic medication use during pregnancy (IPT: 25.5% and EUC: 21.8%; *χ*2 = 0.19; *p* = 0.659*)*. There was no statistically significant difference in intracranial volume between infants born to mothers in the IPT (M = 552,262 mm³, SD = 80,010) and EUC groups (M = 552,027 mm³, SD = 63,639), t(107) = 0.02, *p* = 0.987, 95% CI [–27,101.8, 27,571.2], *d* = 0.003. See Table 1 for sample baseline characteristics between groups.

**Table 1:** Characteristics of the sample

	IPT group (*n* = 52)	EUC group (*n* = 57)	*p*-value
Maternal characteristics			
Maternal age at recruitment, mean (SD)	29.50 (5.37)	30.39 (6.11)	0.425
Gestational age at recruitment, mean (SD), week	17.71 (4.29)	18.13 (4.81)	0.636
Parity (nulliparous)	26 (50.0%)	17 (29.8%)	0.031
Race and ethnicity			0.339
Asian	0 (0%)	2 (3.5%)	
Black	3 (5.8%)	5 (8.8%)	
Hispanic/Latine	7 (13.5%)	13 (22.8%)	
Multiracial/multiethnic	12 (23.1%)	13 (22.8%)	
White	30 (57.7%)	24 (42.1%)	
Household income to needs ratio (living below 200% of the federal poverty line)	20 (38.5%)	24 (42.1%)	0.699
Medicaid status (enrolled)	27 (51.9%)	29 (50.9%)	0.913
Cohabitating with partner	39 (75.0%)	44 (77.2%)	0.788
Education (highest degree earned)			0.308
High school or less	8 (15.4%)	12 (21.1%)	
Some college	20 (38.5%)	17 (29.8%)	
College degree	14 (26.9%)	22 (38.6%)	
Graduate degree	10 (19.2%)	6 (10.5%)	
MDD diagnosis at recruitment	19 (36.5%)	19 (33.3%)	0.726
EPDS at recruitment, mean (SD)	11.98 (4.88)	11.60 (5.29)	0.695
SCL-20 at recruitment, mean (SD)	27.94 (13.50)	26.72 (15.26)	0.660
**Infant characteristics**			
Neonate sex (female)	27 (51.9%)	31 (54.4%)	0.797
Gestational age at birth, mean (SD), week	38.55 (1.56)	38.77 (1.71)	0.487
Postconceptional age at scan, mean (SD), week	45.00 (3.05)	44.60 (2.67)	0.466
Intracranial volume (mm³)	552,262 (80,010)	552,027 (63,639)	0.987

Independent sample t-tests are used for continuous measures. Fisher’s exact tests are used in the contrast of multiple categories of race to accommodate low cell size. A Chi-square test with three degrees of freedom is used for the education contrast. All other contrasts use a Chi-square test with one degree of freedom.

### Infant amygdala and hippocampal volume

**Fig. 3:**
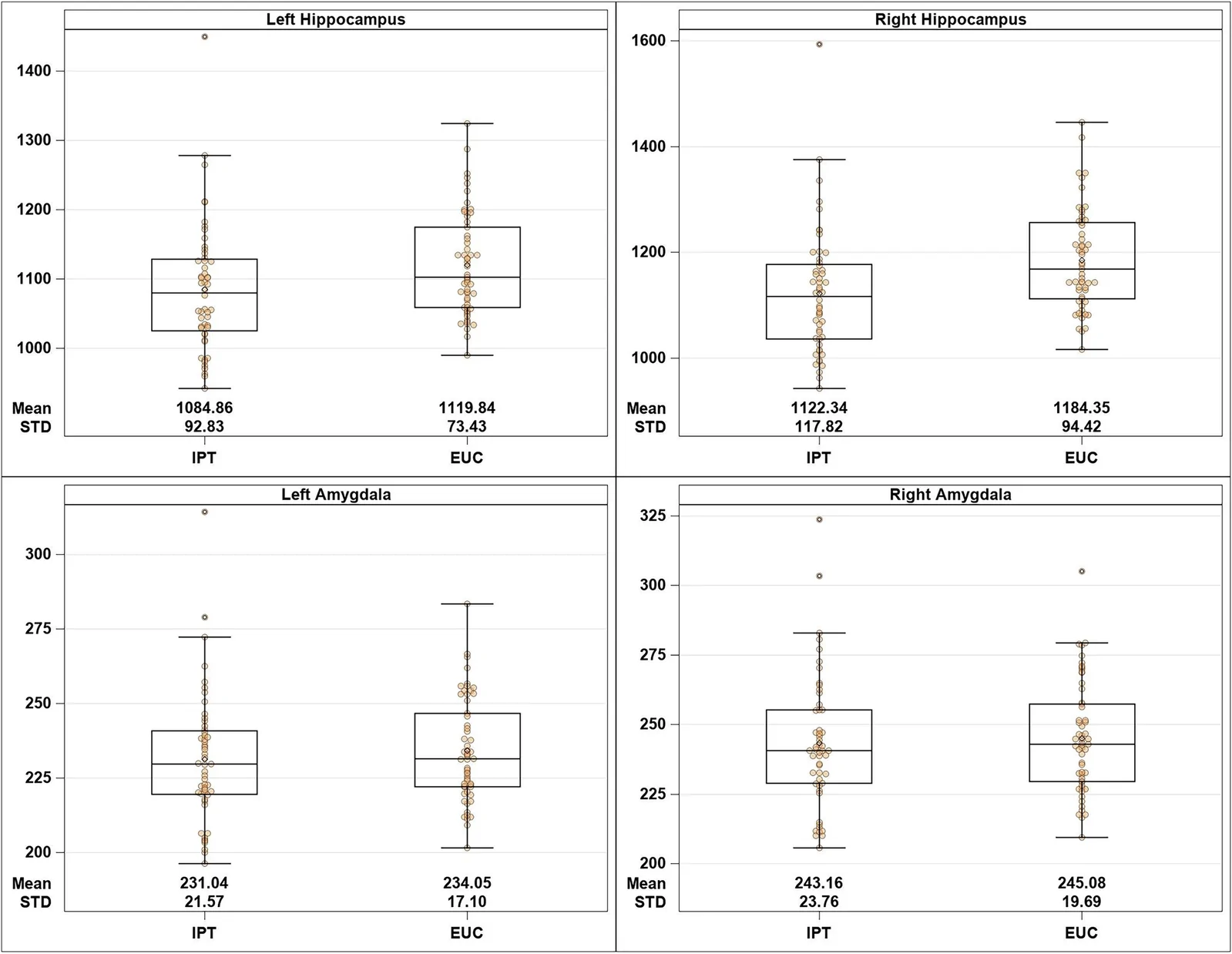
Hippocampal and amygdala volume in infants of mothers randomized to IPT vs. EUC. Top panel: Infants of mothers randomized to the IPT group (*n* = 52) had significantly lower right hippocampal volume than those in the EUC group (*n* = 57; *t*(103) = −3.13, *p* = 0.002, *d* = 0.62, 95% CI [0.23–1.00]). No statistically significant group differences were observed in left hippocampal volume *t*(103) = −1.41, *p* = 0.16, *d* = 0.28, 95% CI [−0.10 - +0.66]. Bottom panel: No statistically significant group differences were observed in amygdala volume (left amygdala: *t*(103) = −0.70, *p* = 0.49, d = 0.14, 95% CI [−0.24 - +0.52]; and right amygdala: *t*(103) = −0.21, *p* = 0.83, d = 0.04, 95% CI [−0.34 - +0.42]). Group comparisons were performed using general linear models with two-sided tests. Bonferroni correction was applied across the four regions of interest. Boxes represent the interquartile range (IQR), center lines indicate the median, whiskers extend to the minimum and maximum within 1.5 × IQR, and circles represent individual datapoints. Plotted values are adjusted for parity, sex, intracranial volume, and postconceptional age at scan. See Supplementary Fig. 2 for plots with unadjusted values.

There was a significant difference in right hippocampal volume between infants of mothers in the IPT and EUC groups (*t*(103)= −3.13, *p* = 0.002, *d* = 0.62, 95% CI [0.23–1.00]) such that infants of participants randomized to IPT had lower right hippocampal volume compared to infants in the EUC group (see Fig. 3). Intervention comparisons for the left hippocampus and bilateral amygdala volumes were not statistically significant; left hippocampus: t(103) = −1.41, *p* = 0.161, *d* = 0.28, 95% CI [−0.10 - +0.66]; right amygdala: t(103) = −0.21, *p* = 0.834, *d* = 0.04, 95% CI [−0.34 - +0.42]; and left amygdala: t(103) = −0.70, *p* = 0.487, *d *= 0.14, 95% CI [−0.24 - +0.52]; see Supplementary Table 2 for full model results). Exclusion of two values that were ±3 standard deviations for the right hippocampus and one value for the left hippocampus did not alter the effect size or significance of these analyses (see Supplementary Table 3 for details). Model-based residuals indicated no deviation from multivariate normality, as assessed by the Shapiro-Wilk test^48^ (SW range = 0.967–0.988, *p*-value range = 0.1–0.49). Post hoc power analyses conducted using the *pwr* package in R indicated >85% power to detect an effect size of d = 0.6. Sensitivity analyses demonstrated that the intervention effects on hippocampal and amygdala volume remained consistent with the original model after additionally covarying for maternal health, maternal pre-pregnancy BMI, obstetric complications, and infant birthweight (Supplementary Table 4) as well as psychotropic medication use (see Supplementary Table 5). Additionally, intervention effects were also robust when covarying for postnatal EPDS (Supplementary Table 6) and imaging quality (Supplementary Table 7).

### Secondary analyses: maternal mental health trajectories

Depressive symptom slopes across pregnancy, based on repeated SCL-20 and EPDS assessments, were not statistically significantly associated with infant hippocampal or amygdala volume. Similarly, there were no significant associations between STAI and PSS slope across pregnancy and infant hippocampal or amygdala volume (see Supplementary Tables 8.1–8.4 for full model results).

### Secondary analyses: sex as a moderator

The intervention group by sex interaction was not statistically significant for hippocampal (all ps > 0.679) or amygdala (all ps > 0.632) volume (see Supplementary Table 9 for full models).

### Secondary analyses: infant negative emotionality

Partial correlations, conducted with left and right amygdala and hippocampal volumes and infant negative emotionality, showed that right hippocampal volume (*r* = 0.19, *p* = 0.045) and left hippocampal volume (*r* = 0.32, *p* < .001) were positively associated with negative emotionality. No statistically significant associations were found for right amygdala volume (*r* = 0.08, *p* = 0.411) or left amygdala volume (*r* = 0.002, *p* = 0.998).

## Discussion

Prenatal maternal depression is one of the most common perinatal complications with profound implications for both maternal and child well-being^49^. Pregnant individuals participated in an RCT that reduced depression (symptoms and disorder) by the end of pregnancy for participants randomized to IPT, as the active intervention group, compared to EUC. Here we present findings from an observational follow-up of infants born to mothers who participated in this RCT^50^. Findings from our study demonstrate that infants of mothers randomized to the IPT treatment group had significantly lower right hippocampal volume compared to infants of mothers in the EUC group. Further, lower hippocampal volume, assessed at 6-weeks of age, was associated with lower infant negative emotionality, as observed via a standardized observational task at 6 months of age.

Leveraging the randomized design of the parent clinical trial, these findings show that infants of mothers randomized to the IPT group had altered early hippocampal volume compared to infants of mothers in the EUC group. Specifically, group differences were observed such that infants of mothers who received IPT had significantly *smaller* right hippocampal volume compared to EUC. To test the functional correlates of hippocampal volume ascertained at 6-weeks of age, we examined links with negative emotionality assessed via behavioral observation at 6 months of age. Findings showed that *smaller* neonatal hippocampal volume was associated with *lower* subsequent infant negative emotionality. These data provide evidence that neonatal hippocampal volume may be linked to prenatal maternal IPT treatment and have implications for emerging emotional development. However, the long-term developmental significance and biological mechanisms underlying these differences remain untested.

Findings from the present investigation are consistent with the possibility that the intervention may attenuate accelerated maturation. As subcortical brain volume increases from infancy through childhood and adolescence^51–53^, it has been hypothesized that early adversity (including maternal mental illness) leads to accelerated maturation^41,54^ (i.e., greater volume earlier in development). While potentially adaptive, such rapid development of subcortical regions may also limit formative periods of plasticity and increase susceptibility to mental health disorders^41,42^. Relatively few studies assessing maternal prenatal depression have evaluated this question. Prior correlational studies in infants link higher prenatal maternal depression to larger hippocampal volume in 2- to 6-week-olds^28^ and subcortical brain volume in infants (ages 3-6 months)^27^, supporting the accelerated maturational hypothesis. Further evidence for an accelerated pattern of brain development is observed in a study linking maternal prenatal depression to altered resting state connectivity^55^. However, findings across the infant literature are inconsistent. One study found a sex-specific effect whereby prenatal depression was associated with *smaller* amygdala volumes in males only^29^, while others found null effects with amygdala and hippocampal volume^38–40^. Taken together, this small infant literature does not yield consensus regarding the impact of prenatal maternal mental health on offspring brain development, highlighting the need for experimental studies to clarify these associations.

While group differences were found within the right hippocampus and not the left, the laterality of these findings should be interpreted with caution, as the pattern of association with prenatal maternal depression exposure was consistent across both the left and right hippocampus. Further evaluation with a higher-powered study represents an avenue for future research. Additionally, group differences were not observed in infant amygdala volume. In correlational work, prenatal depression exposure is associated with amygdala volume in infancy in some^28^, but not all, studies^38,39^. Although we did not observe group differences in amygdala volume in infancy, it is possible that with continued development of the amygdala over the postnatal period, associations with maternal prenatal intervention may emerge^33^. Additional studies are needed to better understand the impact of maternal depression exposure on brain development from infancy through childhood and adulthood^35,56,57^.

The present findings underscore the importance of the intrauterine environment and the intergenerational associations between intervening during pregnancy and offspring brain development. We find prenatal group assignment to IPT versus EUC predicted infant hippocampal volume. Although it is plausible that experiences in the early postpartum period related to treatment group contributed to the observed associations, postnatal maternal depression symptoms did not predict infant hippocampal or amygdala volume, and covarying postnatal maternal depression did not alter the pattern of findings. Notably, the pathways tested within the current study, namely symptoms of depression and anxiety as well as perceived stress, were not significantly associated with infants’ amygdala or hippocampal volume. It may be surprising that maternal prenatal depression trajectories were not associated with amygdala or hippocampal volume. It is possible that the current study was underpowered to detect such effects. Alternatively, it is plausible that in addition to the effects of treatment on depression, IPT, as an efficacious psychosocial intervention, altered pregnant individuals’ biological processes (e.g., HPA axis signaling, inflammatory processes), health behaviors (e.g., sleep, diet, and physical activity) and/or behavioral skill development (e.g., communication and social support) that then impacted fetal development. In terms of biological pathways, disruption to the maternal-placental-fetal HPA axis during gestation alters production of stress-responsive hormones, including cortisol and placental corticotropin-releasing hormone (for review, see ref. ^58^). As these hormones cross the blood-brain barrier, they influence many processes critical to fetal brain maturation, including neuronal dendritic growth, myelination, and neurogenesis^59,60^. Depression is also associated with increased inflammation and altered cytokine levels^61–63^, which have been linked to child mental health^64–66^ and brain development^67^. It is also plausible that IPT alters maternal health behaviors, including sleep, diet, and physical activity^3,68^. Alteration of any of these processes would be expected to have downstream impacts on the developing fetal brain and behavior^42,69–71^. Finally, IPT targets social skill development, and improvement of social skills can both increase social support^72^ and decrease sources of stress related to interpersonal relationships^73,74^. The present study identifies important impacts of randomization on infant brain development. Future work is needed to determine potential mechanisms underlying the effect of IPT treatment on infant developmental outcomes.

This study has several strengths and limitations. First, the randomized design of the parent clinical trial provides a unique opportunity to evaluate infant brain development among offspring born to pregnant participants assigned to prenatal IPT intervention versus EUC. Use of an RCT design more rigorously controls for confounding factors relative to prior correlational research^2,3^. Second, the longitudinal design with neonatal imaging and infant behavioral assessments at 6 months of age enables assessment of the functional implications of subcortical volume. Third, the inclusion of a racially, ethnically, and socioeconomically diverse population enhances the generalizability of study findings. However, data were collected in one U.S. urban area and only in English. Future work should evaluate the benefits of depression reduction across multiple contexts and cultures. Fourth, MRI scans were obtained in young infants, which reduces the potential of confounding effects of postnatal environmental exposures (e.g., parenting influences or nutritional exposure) and increases the likelihood that these effects are due to prenatal pathways. Further, covarying postnatal depression did not alter study findings. However, only one MRI scan was conducted; future work should include a longitudinal assessment of volumetric differences to better understand how prenatal maternal depression intervention influences the rate and trajectory of brain maturation^53,75^. We were underpowered to test the moderating role of sex, and it is plausible that sex differences will emerge with longer-term developmental follow-up. As prior research has identified sex differences in response to prenatal adversity^45,76^, future studies with larger samples and extended follow-up are needed. Finally, accumulating evidence suggests that sociocontextual factors, including experiences of poverty and broader structural stressors, influence maternal mental health during pregnancy^77,78^ and infant neurodevelopment^79,80^. The current IPT active intervention was designed to meet the needs of pregnant individuals experiencing sociocontextual stressors^81^. Future research may investigate how contextual factors amplify or attenuate treatment effects and will help to tailor interventions for those facing the greatest adversity.

Maternal prenatal depression is amenable to treatment and thus is an actionable target for improving both maternal and child health. Study findings support the hypothesis that receiving IPT, as an efficacious prenatal depression intervention, is associated with differences in early offspring structural brain development. The hippocampus is a stress-sensitive region that is implicated in the emergence of subsequent psychopathology^82^. We found that neonatal hippocampal volume was associated with infant negative emotionality, assessed via behavioral observation, at 6 months. This suggests there may be functional implications of alterations to hippocampal volume. An important future direction is understanding the extent to which these group differences in offspring subcortical brain volume during infancy persist across development and predict susceptibility to psychopathology later in development. Exploring how these early structural changes predict later behavioral outcomes beyond infancy will provide valuable insights into early identification and intervention strategies that could mitigate mental health risks across generations. Ultimately, these results highlight the potential of a non-invasive, relatively low-cost prenatal psychotherapy intervention^81^ to positively influence both maternal well-being and developmental trajectories in offspring, underscoring the value of preventive mental health strategies during pregnancy.

## Methods

### Study design

This study is an observational follow-up of infants born to participants in the Care Project, a randomized clinical trial of a prenatal depression intervention (ClinicalTrials.gov; NCT03011801). The parent trial recruited 234 pregnant individuals, primarily from obstetric clinics at two major medical centers in the Denver, Colorado, area. The Colorado Multiple Institutional Review Board (COMIRB) and the University of Denver Institutional Review Boards for the Protection of Human Subjects approved all study protocols. Participants provided written and informed consent, and the study was conducted in accordance with the ethical principles of the Declaration of Helsinki. Participants completed interviews and questionnaires across pregnancy to assess prenatal depressive symptoms and disorder, the trial’s primary and secondary endpoints. Infant neuroimaging and behavioral assessments were subsequently conducted as part of an observational follow-up of offspring born to participants in the parent trial. Neonatal amygdala and hippocampal volume were assessed via structural magnetic resonance imaging (MRI) during natural sleep at 44.8 weeks’ postconceptional age ( ~ 6 weeks old) on average. Infant negative emotionality was assessed via behavioral observation at 6 months (see Supplementary Fig. 1 for study timeline). Participants were compensated for completing study assessments, including the prenatal questionnaires, the infant MRI visit, and the 6-month infant behavioral assessment.

### Participants

Recruitment for the parent RCT began in July 2017, and participants were enrolled from August 10, 2017, to September 8, 2021. Eligibility criteria included being 18–45 years of age, English-speaking, 25 weeks gestational age (GA) or less, singleton pregnancy, and endorsing elevated depression symptoms when screened as part of standard of care (Edinburgh Postnatal Depression Scale; EPDS score ≥10). Exclusion criteria included current illicit drug or methadone use and major health conditions requiring invasive treatments (e.g., dialysis, blood transfusions, chemotherapy). Additional assessment for eligibility was performed at baseline to exclude 1) current or past psychosis or mania and 2) currently receiving cognitive behavioral therapy or interpersonal psychotherapy. Additional exclusion criteria for the offspring follow-up included a) major fetal or chromosomal anomalies (*n* = 1) or b) any infant MRI contraindications (*n* = 1) (e.g., baby on supplemental oxygen). The final analytic sample consisted of 109 participants; see Fig. 1 for the CONSORT Diagram detailing participant flow, including reasons for loss to follow-up (e.g., missed due to the COVID-19 shutdown) and the exclusion of certain imaged infants (e.g., failed quality control procedures).

### Randomization and masking

Eligible participants in the parent randomized clinical trial^44,50^ were assessed at baseline for depression and then randomly assigned via parallel assignment to Interpersonal Psychotherapy (IPT) or Enhanced Usual Care (EUC) using a computer-generated random numbers sequence. Participants were randomized in blocks of two and stratified on current major depressive disorder (MDD), gestational age (GA) (above or below 15 weeks), and Medicaid status for each study arm. Research staff masked to condition performed interviews for depression assessment, medical record reviews, and collected sociodemographic and obstetric data.

### Procedures

#### Intervention conditions

The depression intervention, MOMCare, is a form of brief IPT delivered within a collaborative care model^81^. The intervention effectively reduced depressive symptoms and rates of major depressive disorder during pregnancy^50^. Brief IPT focuses on improving interpersonal relationships and social functioning to reduce depression symptoms^83^. MOMCare consisted of 8 weekly, 50-minute individual sessions. The enhanced usual care (EUC) group received standard of care through their provider, which was then enhanced through the inclusion of a 1-hour psychoeducation session with a study team clinician and ongoing monitoring throughout the prenatal period. All maternal and infant assessments were performed by evaluators blinded to condition.

#### Maternal depression

Participants completed self-report depression measures via REDCap three to five times across pregnancy: 16.41 (4.08) weeks (prior to intervention), followed by subsequent assessments at: 22.10 (4.01) weeks, 25.61 (4.08) weeks, 29.42 (4.18) weeks (near the end of active IPT), and 35.73 (1.58) weeks (end of gestation). The EPDS was administered at 6 weeks postpartum, concurrent with the MRI visit.

Edinburgh postnatal depression scale (EPDS)^84^, a 10-item measure employing a 0-3 Likert scale, was administered to assess depression symptoms five times across pregnancy and concurrent with the MRI assessment postnatally. Total scores range from 0-30, with scores ≥10 suggesting probable depression^84^. The EPDS has robust reliability and validity during pregnancy^85^. Internal consistency for the EPDS was greater than 0.8 across all five time points.

Symptom Checklist (SCL-20) is a 20-item measure derived from the Symptom Checklist-90-R item scale 8^4^. The SCL-20 was administered three times across pregnancy (baseline, 29-, and 35-weeks’ GA). Responses were recorded on a 0-4 Likert scale and summed to generate a total score, ranging from 0–80^86,87^. Internal consistency was excellent (α > 0.9 across all three timepoints).

#### Maternal anxiety and perceived stress

In addition to measures of depression, participants also completed the Spielberger State-Trait Anxiety Inventory (STAI)^88^ and the Perceived Stress Scale (PSS)^89^, which were administered at the same time as the depression assessments.

State-Trait Anxiety Inventory (STAI)^88^. The 20-item State Anxiety subscale of the State–Trait Anxiety Inventory was administered to assess generalized anxiety three times across pregnancy (baseline, 29-, and 35-weeks’ GA). Participants reported how they had felt over the past week, including the day of assessment. Example items include “I am tense” and “I am worried.” Items were rated on a 4-point Likert scale, with higher scores indicating greater distress.

Perceived Stress Scale (PSS)^89^ assesses subjective perceptions of recent stressful life events using 10 items, rated on a 0 (never) to 4 (very often) scale. Example items include, “In the last month, how often have you been upset because of something that happened unexpectedly”, and “In the last month, how often have you felt nervous and stressed”. The PSS was assessed five times across pregnancy.

#### Infant negative emotionality

Behavioral assessment of infant negative emotionality was assessed using the Robot Behavioral Task from the Laboratory Temperament Assessment Battery (Lab-TAB)^90^. The Robot Behavioral Task is a standardized and validated measure involving exposure to a robot that moves and makes noises. Infant responses to the robot were video-recorded and subsequently coded for intensity of facial negative affectivity (0–4) and distress vocalizations (0–5). Scores across facial and vocalization intensity were standardized and averaged to create a composite score of infant negative emotionality, with higher scores indicating higher negative emotionality. Twenty percent of recordings were double-coded by independent raters, with excellent interrater reliability (0.94).

#### Gestational dating and medical record review

Two research staff blinded to participants’ treatment condition performed medical record reviews to obtain gestational dating. Gestational age (GA) and postconceptional age at scan (PCA) were calculated from medical records review, applying American College of Obstetricians and Gynecologists (ACOG) guidelines of first-trimester ultrasound and/or date of the last menstrual period^91^. Postconceptional age at scan was calculated as the sum of gestational age at birth and the infant’s chronological age since birth (at the time of the MRI scan). Additional obstetric information, including parity and obstetric complications, maternal height and weight, and infant sex and birthweight, was obtained from medical records. Maternal-fetal obstetric complications scores were calculated by adding the number of pregnancy and birth-related complications, including prenatal infection, pregnancy-induced hypertension, gestational diabetes, anemia, and intrauterine growth restriction^42,92^.

#### Demographic characteristics

Research staff blinded to the condition collected participant self-reported sociodemographic characteristics, including birthdate, socioeconomic status, cohabitation with a partner, marital status, educational attainment, and race and ethnicity. Income-to-needs ratio (INR) was calculated by dividing the total reported household income for the past year by the federal poverty threshold for that year corresponding to the number of persons living in the household specified by the U.S. Census Bureau.

#### Magnetic resonance imaging acquisition

Infants were scanned during natural sleep. A Siemens Skyra 3 T MRI system equipped with a 20-channel head coil at the Brain Imaging Center at the University of Colorado Anschutz Medical Campus was used. Before scanning, infants were fed, swaddled, and placed into the scanner with their head secured within a vacuum-fixation device to limit motion. Infants wore earplugs and headphones to limit the acoustic noise of the scan. Infants were monitored by a research staff member who was in the scanner with the infant for the duration of the scan.

T1-weighted (T1w) images were obtained using a three-dimensional magnetization-prepared rapid gradient echo sequence (repetition time = 1900 ms; echo time = 3.07 ms; inversion time = 900 ms; flip angle 9°; 4 min 26 s) and T2-weighted (T2w) images were obtained with a 3D fast turbo spin echo sequence (repetition time 3200 ms; echo time = 408 ms; flip angle var; 4 min 43 s). The spatial resolution was a 0.82 × 0.82 × 0.8 mm voxel for T1w and 0.86 × 0.86 × 0.8 mm voxel for T2w.

#### Magnetic resonance image processing

Image quality control (QC) was assessed using a four-point scale (0–3)^93^ adapted in-house for infant scanning. The criteria for exclusion were a QC score of 0, indicating artifact contamination, rendering the image processing unreliable mainly due to subject motion. Three scans were excluded based on failed T1w image quality. For participants who had artifact contamination in T2w image, T2 data were imputed from a passing T1w image (*n* = 10)^94^.

The T1w and T2w brain images were corrected for intensity non-uniformity via N4^95^, rigidly transformed to a prior pediatric neonate atlas in stereotaxic space^96^. Brain masking was performed via the 3D UNet-based brain masking tool in ANTSPyNet^97^ using both T1w and T2w images jointly, including also extra-axial cerebrospinal fluid spaces in the brain mask. All brain masks were corrected manually in ITK-SNAP^98^.

Tissue segmentation, regional parcellation, as well as hippocampus and amygdala segmentation were performed using a multi-modality (T1w and T2w), multi-atlas segmentation workflow with the in-house, open-source MultiSegPipeline software, which employs atlas-registration and label fusion from the ANTs toolset^97^. Hippocampal and amygdala regions were defined as in ref. ^99^ (see Fig. 2). Total intracranial volume was calculated as the sum of the brain tissue volumes of gray matter, white matter, and cerebrospinal fluid. The segmentation quality of all images was visually assessed and rated using a four-point scale (0–3) for anatomical accuracy as in refs. ^71,100^. Volumetric measurements rated as 0 (failed segmentation) were excluded from the analysis. No scans were removed based on segmentation quality for either the hippocampus or the amygdala. Outliers in bilateral hippocampal and amygdala volume data were defined as values exceeding three standard deviations above or below the mean.

### Statistical analysis

All analyses were conducted in SAS version 9.4 and included participants with brain outcome measures (*n* = 109). We first examined potential selection bias for those included in the analytic sample compared to the total 234 randomized participants from the full RCT and found no demographic or clinical differences (see Supplementary Table 1). Table 1 provides descriptive statistics for IPT and EUC groups in our analytic sample. The RCT design stratified on gestational age at recruitment, Medicaid status, and MDD diagnosis, and as expected, these variables did not differ between groups. Only parity differed between intervention groups, so it was included as a covariate; no other demographic variables significantly differed between groups (see Table 1). All observed data were used to create a complete dataset (*n* = 109) via Markov chain Monte Carlo imputation methods^101^; this complete dataset was used for analyses.

A general linear model was used to test group differences in IPT versus EUC, in bilateral amygdala and hippocampal volume, covarying for postconceptional age at scan, intracranial volume (ICV), infant sex, and parity. To account for multiple comparisons across the four subcortical regions (bilateral hippocampus and amygdala), a Bonferroni correction was applied (corrected α = .01250), and all statistical tests were two-sided. Cohen’s *d* effect sizes for intervention-group differences were derived from the model-adjusted mean difference in volume between groups. In sensitivity analyses, other potential confounding factors, including maternal physical health (i.e., diagnosis of hypertension and/or diabetes), body mass index (BMI), obstetric complications, infant birthweight, postnatal maternal depression symptoms, psychotropic medication use, and image quality were included as additional covariates.

Secondary analyses examined whether 1) depression trajectory during pregnancy would independently influence infant hippocampal and amygdala volume; 2) sex moderated treatment effects; and 3) neonatal amygdala and hippocampal volume were associated with infant negative emotionality at 6 months. To test whether changes in depressive symptoms across pregnancy were associated with infant amygdala and hippocampal volume, symptom trajectories, assessed via the SCL-20 and EPDS, were entered as separate predictors in the model. In addition to depression, other mental health indicators, including trajectories of maternal anxiety (assessed via the STAI) and stress (assessed via the PSS), were also examined as model predictors. To test for moderation by infant sex, a treatment group × infant sex interaction term was included in the model predicting amygdala and hippocampal volume. Finally, partial correlations adjusting for intracranial volume and postconceptional age at scan were conducted to examine associations between subcortical volume and infants’ observed negative emotionality, assessed via laboratory-based observation at 6 months. Associations are reported as Pearson correlation coefficients (*r*).

### Reporting summary

Further information on research design is available in the Nature Portfolio Reporting Summary linked to this article.

## Supplementary information

Supplementary Information

Reporting Summary

Transparent Peer Review file

## Data Availability

The behavioral and imaging data generated in this study have been deposited in the University of Denver Digital Commons database: https://doi.org/10.56902/Data.Psych.2026.1.

## Code availability

The statistical code used to generate results reported in this study have been deposited in the University of Denver Digital Commons database: https://doi.org/10.56902/Data.Psych.2026.1.
