# Supplementary material for: Infant hippocampal and amygdala volume in an observational follow-up from a randomized clinical trial for maternal prenatal depression
Source: Nat Commun. 2026 Oct 16;17:10649. doi: 10.1038/s41467-026-78276-4 (PMC13675242; doi:10.1038/s41467-026-78276-4)
Supplement: Supplementary file 1 [file 41467_2026_78276_MOESM1_ESM.pdf]

Supplemental Content

Supplemental Table 1. Comparison of the analytic sample with infant brain imaging data (n = 109) and participants without infant brain imaging data (n = 125) from the parent RCT (N = 234).

Supplementary Table 2. Hippocampal and amygdala volume in infants of mothers randomized to IPT vs. EUC.

Supplementary Table 3. Hippocampal and amygdala volume in infants of mothers randomized to IPT vs. EUC, removing outliers.

Supplementary Table 4. Hippocampal and amygdala volume in infants of mothers randomized to IPT vs. EUC, additionally covarying for maternal body mass index (BMI), obstetric complications, diagnosis of diabetes or hypertensive disorders, and infant birthweight.

Supplementary Table 5. Hippocampal and amygdala volume in infants of mothers randomized to IPT vs. EUC, additionally covarying for psychotropic medication use.

Supplementary Table 6. Hippocampal and amygdala volume in infants of mothers randomized to IPT vs. EUC, additionally covarying for postnatal EPDS.

Supplementary Table 7. Hippocampal and amygdala volume in infants of mothers randomized to IPT vs. EUC, additionally covarying for imaging quality.

Supplementary Table 8.1–8.4. Association between maternal prenatal mental health symptom trajectory and infant hippocampal and amygdala volume:

- **8.1.** Edinburgh Postnatal Depression Scale (EPDS)
- **8.2.** Symptom Checklist-20 (SCL-20)
- **8.3.** State-Trait Anxiety Inventory (STAI)
- **8.4.** Perceived Stress Scale (PSS)

Supplementary Table 9. Maternal IPT vs. EUC group by infant sex interaction on hippocampal and amygdala volume.

Supplemental Figure 1. Timeline of the parent randomized clinical trial and observational infant follow-up.

Supplemental Figure 2. Infant hippocampal and amygdala volume by maternal IPT vs. EUC group, unadjusted for covariates (raw values).

This supplemental material has been provided by the authors to give readers additional information about their work.

Supplementary Table 1

Comparison of the analytic sample with infant brain imaging data (n = 109) and participants without infant brain imaging data (n = 125) from the parent RCT (N = 234).

	Participants without brain imaging data (n=125)	Analytic sample with brain imaging data (n=109)	p-value
Maternal age at recruitment, mean (SD)	29.73 (6.00)	29.96 (5.76)	0.761
Gestational age at recruitment, mean (SD), week	18.27 (4.32)	17.93 (4.55)	0.562
Parity (nulliparous)	65 (52.0%)	43 (39.4%)	0.055
Race and ethnicity			0.248
Asian	8 (6.4%)	2 (1.8%)	
Black	13 (10.4%)	8 (7.3%)	
Hispanic/Latine	23 (18.4)	20 (18.3%)	
Native Hawaiian/Pacific Islander	1 (0.8%)	0 (0%)	
Multiracial/multiethnic	33 (26.4%)	25 (22.9%)	
White	47 (37.6%)	54 (49.5%)	
Household income to needs ratio (living below 200% of the federal poverty line)	50 (40.0%)	44 (40.4%)	0.955
Medicaid status (enrolled)	63 (50.4%)	56 (51.4%)	0.882
Cohabitating with partner	91 (72.8%)	83 (76.1%)	0.559
Education (highest degree earned)			0.135
High school or less	38 (30.4%)	20 (18.3%)	
Some college	33 (26.4%)	37 (33.9%)	
College degree	33 (26.4%)	36 (33.0%)	
Graduate degree	21 (16.8%)	16 (14.7%)	
MDD diagnosis at recruitment	48 (38.4%)	38 (34.9%)	0.576
MDD diagnosis at end of pregnancy	21 (16.8%)	17 (15.6%)	0.803
EPDS at recruitment, mean (SD)	11.94 (4.80)	11.78 (5.08)	0.809
SCL-20 at recruitment, mean (SD)	28.42 (14.05)	27.30 (14.40)	0.548

Values presented as mean (SD) or n (%). Independent sample t-tests are used for continuous measures. Fisher's exact tests are used in the contrast of multiple categories of race to accommodate low cell size. Chi-square test with three degrees of freedom is used for the education contrast. All other contrasts use a Chi-square test with one degree of freedom.

Supplementary Table 2

Hippocampal and amygdala volume in infants of mothers randomized to IPT vs. EUC.

Brain Region	Predictor	Beta	Std Error	*t*(103)	*p-value*
Right Hippocampus	Intercept	1186.64	14.0588	84.41	<.001
	Parity	-9.6407	10.1502	-0.95	0.344
	Sex	-10.7994	10.8910	-0.99	0.324
	Age at Scan	-15.3396	14.8594	-1.03	0.304
	ICV	119.14	15.5124	7.68	<.001
	Intervention group	-63.9078	20.4321	-3.13	0.002
Left Hippocampus	Intercept	1119.94	16.7160	67.00	<.001
	Parity	-0.4502	12.0686	-0.04	0.970
	Sex	-2.2366	12.9495	-0.17	0.863
	Age at Scan	-10.0287	17.6679	-0.57	0.572
	ICV	90.8926	18.4443	4.93	<.001
	Intervention group	-34.2775	24.2939	-1.41	0.161
Right Amygdala	Intercept	244.57	3.4557	70.77	<.001
	Parity	-0.1375	2.4949	-0.06	0.956
	Sex	8.7880	2.6770	3.28	0.001
	Age at Scan	-9.5148	3.6524	-2.61	0.011
	ICV	22.3769	3.8129	5.87	<.001
	Intervention group	-1.0527	5.0222	-0.21	0.834
Left Amygdala	Intercept	234.24	3.1276	74.89	<.001
	Parity	-1.1068	2.2581	-0.49	0.625
	Sex	3.6196	2.4229	1.49	0.138
	Age at Scan	-2.8949	3.3058	-0.88	0.383
	ICV	19.8964	3.4510	5.77	<.001
	Intervention group	-3.1733	4.5455	-0.70	0.487

A general linear model was used to test group differences in IPT and EUC, in bilateral amygdala and hippocampal volume, covarying for postconceptional age at scan, intracranial volume (ICV), infant sex, and parity. To account for multiple comparisons across the four subcortical regions (bilateral hippocampus and amygdala), a Bonferroni correction was applied (corrected $\alpha = .01250$), and all statistical tests were two-sided. Predictors are standardized; therefore, betas represent on-average increase in volume (mm^3) per standard deviation change in the predictor. The intervention group beta represents the on-average difference between IPT and EUC with negative values indicating IPT less than EUC and positive values indicating IPT greater than EUC.

Supplementary Table 3

Hippocampal and amygdala volume in infants of mothers randomized to IPT vs. EUC, excluding outliers.

Brain Region	Predictor	*F*	*p-value*
Right Hippocampus (n=107)	Parity	0.45	0.506
	Sex	0.45	0.506
	Age at scan	1.33	0.251
	ICV	45.98	<.001
	Intervention group	8.80	0.004
Left Hippocampus (n=108)	Parity	0.04	0.847
	Sex	0.01	0.904
	Age at scan	0.54	0.466
	ICV	22.43	<.001
	Intervention group	1.34	0.250

A general linear model was used to test group differences between IPT and EUC in bilateral amygdala and hippocampal volume, covarying for postconceptional age at scan, intracranial volume (ICV), infant sex, and parity, after excluding outliers (>3 SD from the mean). To account for multiple comparisons across the four subcortical regions (bilateral hippocampus and amygdala), a Bonferroni correction was applied (corrected α = 0.0125), and all statistical tests were two-sided. Two outliers were removed for right hippocampus and one outlier was removed for the left hippocampus; there were no outliers for bilateral amygdala.

Supplementary Table 4

Hippocampal and amygdala volume in infants of mothers randomized to IPT vs. EUC, additionally covarying for maternal body mass index, obstetric complications, diabetes or hypertensive disorders and birthweight.

Brain Region	Predictor	*F*(99)	*p-value*
Right Hippocampus	Parity	1.04	0.309
	Sex	0.66	0.419
	Age at scan	0.93	0.337
	ICV	46.78	<.001
	Maternal BMI	0.57	0.452
	Hypertension/Diabetes	0.18	0.676
	OB complications	0.02	0.881
	Birthweight	2.77	0.099
	Intervention group	8.60	0.004
Left Hippocampus	Parity	0.01	0.930
	Sex	0.00	0.986
	Age at scan	0.33	0.567
	ICV	20.30	<.001
	Maternal BMI	0.91	0.343
	Hypertension/Diabetes	0.00	0.976
	OB complications	0.35	0.554
	Birthweight	0.56	0.455
	Intervention group	1.52	0.221
Right Amygdala	Parity	0.01	0.944
	Sex	9.96	0.002
	Age at scan	5.87	0.017
	ICV	26.72	<.001
	Maternal BMI	0.35	0.555
	Hypertension/Diabetes	0.18	0.672
	OB complications	0.33	0.568
	Birthweight	0.23	0.633
	Intervention group	0.09	0.769
Left Amygdala	Parity	0.13	0.719
	Sex	1.97	0.164
	Age at scan	0.26	0.613
	ICV	24.02	<.001
	Maternal BMI	0.04	0.837
	Hypertension/Diabetes	0.54	0.466
	OB complications	0.00	0.982
	Birthweight	0.44	0.509
	Intervention group	0.26	0.609

A general linear model was used to test group differences between IPT and EUC in bilateral amygdala and hippocampal volume, covarying for postconceptional age at scan, intracranial volume (ICV), infant sex, parity, maternal body mass index (BMI), obstetric complications, maternal hypertension and/or diabetes, and infant birthweight. To account for multiple comparisons across the four subcortical regions (bilateral hippocampus and amygdala), a Bonferroni correction was applied (corrected $\alpha = 0.0125$), and all statistical tests were two-sided.

Supplementary Table 5

Hippocampal and amygdala volume in infants of mothers randomized to IPT vs. EUC, additionally covarying for psychotropic medication use.

Brain Region	Predictor	*F*(95)	*p-value*
Right Hippocampus	Parity	0.60	0.441
	Sex	0.81	0.371
	Age at scan	0.82	0.368
	ICV	52.37	< .001
	Psychotropic med use	0.70	0.405
	Intervention group	7.71	0.007
Left Hippocampus	Parity	0.02	0.894
	Sex	0.00	0.987
	Age at scan	0.36	0.549
	ICV	22.78	< .001
	Psychotropic med use	0.93	0.337
	Intervention group	1.94	0.167
Right Amygdala	Parity	0.11	0.745
	Sex	12.35	0.001
	Age at scan	6.39	0.013
	ICV	33.31	< .001
	Psychotropic med use	2.38	0.126
	Intervention group	0.00	0.971
Left Amygdala	Parity	0.04	0.852
	Sex	2.94	0.090
	Age at scan	0.87	0.354
	ICV	32.39	< .001
	Psychotropic med use	1.38	0.243
	Intervention group	0.38	0.537

A general linear model was used to test group differences between IPT and EUC in bilateral amygdala and hippocampal volume, covarying for postconceptional age at scan, intracranial volume (ICV), infant sex, parity, and prenatal psychotropic medication use. To account for multiple comparisons across the four subcortical regions (bilateral hippocampus and amygdala), a Bonferroni correction was applied (corrected $\alpha = 0.0125$), and all statistical tests were two-sided. n=102.

Supplementary Table 6

Hippocampal and amygdala volume in infants of mothers randomized to IPT vs. EUC, additionally covarying for postnatal EPDS.

Brain Region	Predictor	*F*(102)	*p-value*
Right Hippocampus	Parity	0.89	0.347
	Sex	0.95	0.333
	Age at scan	1.01	0.317
	ICV	58.10	<.001
	Postnatal EPDS	0.00	0.968
	Intervention group	9.32	0.003
Left Hippocampus	Parity	0.00	0.998
	Sex	0.05	0.819
	Age at scan	0.40	0.528
	ICV	24.30	<.001
	Postnatal EPDS	0.21	0.645
	Intervention group	1.64	0.204
Right Amygdala	Parity	0.00	0.954
	Sex	9.60	0.003
	Age at scan	7.82	0.006
	ICV	35.60	<.001
	Postnatal EPDS	1.76	0.188
	Intervention group	0.00	0.945
Left Amygdala	Parity	0.26	0.612
	Sex	2.28	0.134
	Age at scan	0.67	0.417
	ICV	32.58	<.001
	Postnatal EPDS	0.07	0.789
	Intervention group	0.54	0.464

A general linear model was used to test group differences between IPT and EUC in bilateral amygdala and hippocampal volume, covarying for postconceptional age at scan, intracranial volume (ICV), infant sex, parity, and postnatal maternal depressive symptoms (Edinburgh Postnatal Depression Scale [EPDS]). To account for multiple comparisons across the four subcortical regions (bilateral hippocampus and amygdala), a Bonferroni correction was applied (corrected $\alpha = 0.0125$), and all statistical tests were two-sided.

Supplementary Table 7

Hippocampal and amygdala volume in infants of mothers randomized to IPT vs. EUC, additionally covarying for image quality.

Brain Region	Predictor	*F*(102)	*p-value*
Right Hippocampus	Parity	0.93	0.338
	Sex	1.02	0.316
	Age at scan	1.04	0.310
	ICV	56.37	<.001
	Image quality	0.26	0.609
	Intervention group	9.51	0.003
Left Hippocampus	Parity	0.00	0.960
	Sex	0.04	0.852
	Age at scan	0.31	0.578
	ICV	23.11	<.001
	Image quality	0.14	0.705
	Intervention group	1.91	0.170
Right Amygdala	Parity	0.00	0.994
	Sex	11.49	0.001
	Age at scan	7.09	0.009
	ICV	37.53	<.001
	Image quality	3.27	0.074
	Intervention group	0.10	0.757
Left Amygdala	Parity	0.20	0.657
	Sex	2.41	0.124
	Age at scan	0.81	0.370
	ICV	34.99	<.001
	Image quality	1.77	0.187
	Intervention group	0.60	0.442

A general linear model was used to test group differences between IPT and EUC in bilateral amygdala and hippocampal volume, covarying for postconceptional age at scan, intracranial volume (ICV), infant sex, parity, and MRI image quality. To account for multiple comparisons across the four subcortical regions (bilateral hippocampus and amygdala), a Bonferroni correction was applied (corrected $\alpha = 0.0125$), and all statistical tests were two-sided.

Supplementary Table 8.1

Association between maternal depression symptom trajectory (measured via the EPDS) on hippocampal and amygdala volume.

Brain Region	Predictor	Estimate	SE	*t*(101)	*p-value*
Right Hippocampus	Partial eta EPDS intercept	-0.0131	0.0668	-0.20	0.845
	Partial eta EPDS slope	0.0321	0.0809	0.40	0.692
	Sex	-0.0673	0.0687	-0.98	0.330
	Parity	-0.0643	0.0638	-1.01	0.316
	ICV	0.7256	0.0961	7.55	<.001
	Age at scan	-0.0920	0.0931	-0.99	0.326
	Intervention group	-0.3803	0.1281	-2.97	0.004
Left Hippocampus	Partial eta EPDS intercept	-0.0410	0.0817	-0.50	0.617
	Partial eta EPDS slope	0.1043	0.0990	1.05	0.295
	Sex	-0.0190	0.0841	-0.23	0.821
	Parity	-0.0207	0.0780	-0.27	0.791
	ICV	0.5742	0.1177	4.88	<.001
	Age at scan	-0.0590	0.1140	-0.52	0.606
	Intervention group	-0.1872	0.1568	-1.19	0.235
Right Amygdala	Partial eta EPDS intercept	0.1152	0.0783	1.47	0.144
	Partial eta EPDS slope	0.1376	0.0948	1.45	0.150
	Sex	0.2273	0.0806	2.82	0.006
	Parity	-0.0259	0.0748	-0.35	0.729
	ICV	0.6836	0.1127	6.06	<.001
	Age at scan	-0.3150	0.1092	-2.89	0.005
	Intervention group	0.0146	0.1502	0.10	0.923
Left Amygdala	Partial eta EPDS intercept	0.1123	0.0814	1.38	0.171
	Partial eta EPDS slope	0.0368	0.0986	0.37	0.710
	Sex	0.1013	0.0838	1.21	0.229
	Parity	-0.0423	0.0777	-0.54	0.588
	ICV	0.6890	0.1172	5.88	<.001
	Age at scan	-0.1264	0.1135	-1.11	0.268
	Intervention group	-0.0916	0.1562	-0.59	0.559

Maternal Edinburgh Postnatal Depression Scale (EPDS) intercept and slope across pregnancy were first estimated using hierarchical linear modeling. The resulting estimates were then entered as predictors in a general linear model of bilateral hippocampal and amygdala volume, covarying for postconceptional age at scan, intracranial volume (ICV), infant sex, and parity. All statistical tests were two-sided. Covariates and outcomes are standardized in all models.

Supplementary Table 8.2

Association between maternal depression symptom trajectory (measured via the SCL-20) on hippocampal and amygdala volume.

Brain Region	Predictor	Estimate	SE	*t*(101)	*p-value*
Right Hippocampus	Partial eta SCL20 intercept	0.0168	0.0690	0.24	0.808
	Partial eta SCL20 slope	-0.0435	0.0932	-0.47	0.642
	Sex	-0.0657	0.0673	-0.98	0.331
	Parity	-0.0558	0.0628	-0.89	0.376
	ICV	0.7248	0.0964	7.52	<.001
	Age at scan	-0.0984	0.0929	-1.06	0.292
	Intervention group	-0.4115	0.1341	-3.07	0.003
Left Hippocampus	Partial eta SCL20 intercept	-0.0207	0.0853	-0.24	0.809
	Partial eta SCL20 slope	-0.0582	0.1152	-0.51	0.615
	Sex	-0.0104	0.0833	-0.13	0.901
	Parity	0.0020	0.0777	0.03	0.980
	ICV	0.5677	0.1192	4.76	<.001
	Age at scan	-0.0588	0.1149	-0.51	0.610
	Intervention group	-0.2467	0.1659	-1.49	0.140
Right Amygdala	Partial eta SCL20 intercept	0.1565	0.0805	1.95	0.055
	Partial eta SCL20 slope	0.1675	0.1087	1.54	0.126
	Sex	0.2399	0.0785	3.06	0.003
	Parity	-0.0199	0.0733	-0.27	0.786
	ICV	0.6951	0.1125	6.18	<.001
	Age at scan	-0.3192	0.1084	-2.95	0.004
	Intervention group	0.0546	0.1564	0.35	0.728
Left Amygdala	Partial eta SCL20 intercept	0.1036	0.0841	1.23	0.221
	Partial eta SCL20 slope	-0.0058	0.1136	-0.05	0.959
	Sex	0.1134	0.0821	1.38	0.170
	Parity	-0.0389	0.0766	-0.51	0.612
	ICV	0.6841	0.1176	5.82	<.001
	Age at scan	-0.1247	0.1133	-1.10	0.274
	Intervention group	-0.1095	0.1636	-0.67	0.505

Maternal Depression Symptom Checklist (SCL-20) intercept and slope across pregnancy were first estimated using hierarchical linear modeling. The resulting estimates were then entered as predictors in a general linear model of bilateral hippocampal and amygdala volume, covarying for postconceptional age at scan, intracranial volume (ICV), infant sex, and parity. All statistical tests were two-sided. Covariates and outcomes are standardized in all models.

Supplementary Table 8.3

Association between maternal anxiety symptom trajectory (measured via the STAI) on hippocampal and amygdala volume.

Brain Region	Predictor	Estimate	SE	*t*(101)	*p-value*
Right Hippocampus	Partial eta STAI intercept	-0.0238	0.0743	-0.32	0.749
	Partial eta STAI slope	0.0147	0.0777	0.19	0.851
	Sex	-0.0621	0.0692	-0.90	0.372
	Parity	-0.0599	0.0626	-0.96	0.341
	ICV	0.7198	0.0969	7.43	<.001
	Age at scan	-0.0836	0.0941	-0.89	0.377
	Intervention group	-0.3893	0.1276	-3.05	0.003
Left Hippocampus	Partial eta STAI intercept	-0.0134	0.0916	-0.15	0.884
	Partial eta STAI slope	0.0689	0.0958	0.72	0.474
	Sex	-0.0165	0.0853	-0.19	0.847
	parity	-0.0076	0.0771	-0.10	0.921
	ICV	0.5701	0.1194	4.77	<.001
	Age at scan	-0.0500	0.1160	-0.43	0.667
	Intervention group	-0.2037	0.1572	-1.30	0.198
Right Amygdala	Partial eta STAI intercept	0.1189	0.0875	1.36	0.177
	Partial eta STAI slope	0.0748	0.0915	0.82	0.416
	Sex	0.2299	0.0816	2.82	0.006
	Parity	-0.0083	0.0737	-0.11	0.910
	ICV	0.6857	0.1142	6.01	<.001
	Age at scan	-0.3102	0.1109	-2.80	0.006
	Intervention group	0.0007	0.1503	0.00	0.997
Left Amygdala	Partial eta STAI intercept	0.1031	0.0907	1.14	0.259
	Partial eta STAI slope	0.0857	0.0949	0.90	0.369
	Sex	0.0953	0.0846	1.13	0.262
	Parity	-0.0424	0.0764	-0.55	0.581
	ICV	0.6923	0.1183	5.85	<.001
	Age at scan	-0.1207	0.1150	-1.05	0.296
	Intervention group	-0.0748	0.1558	-0.48	0.632

Maternal State Trait Anxiety Inventory (STAI) intercept and slope across pregnancy were first estimated using hierarchical linear modeling. The resulting estimates were then entered as predictors in a general linear model of bilateral hippocampal and amygdala volume, covarying for postconceptional age at scan, intracranial volume (ICV), infant sex, and parity. All statistical tests were two-sided. Covariates and outcomes are standardized in all models.

Supplementary Table 8.4

Association between maternal perceived stress trajectory (measured via the PSS) on hippocampal and amygdala volume.

Brain Region	Predictor	Estimate	SE	*t*(101)	*p-value*
Right Hippocampus	Partial eta PSS intercept	0.0245	0.0692	0.35	.724
	Partial eta PSS slope	-0.0166	0.0694	–0.24	.811
	Sex	-0.0688	0.0704	–0.98	.331
	Parity	-0.0537	0.0634	–0.85	.399
	ICV	0.7332	0.0974	7.53	<.001
	Age at scan	-0.1055	0.0952	–1.11	.270
	Intervention group	-0.3933	0.1279	–3.07	.003
Left Hippocampus	Partial eta PSS intercept	-0.0095	0.0855	–0.11	.912
	Partial eta PSS slope	0.0281	0.0858	0.33	.744
	Sex	-0.0164	0.0871	–0.19	.851
	Parity	-0.0084	0.0783	–0.11	.914
	ICV	0.5726	0.1203	4.76	<.001
	Age at scan	-0.0563	0.1176	–0.48	.633
	Intervention group	-0.2084	0.1581	–1.32	.191
Right Amygdala	Partial eta PSS intercept	0.0811	0.0815	0.99	.322
	Partial eta PSS slope	-0.0462	0.0818	–0.57	.573
	Sex	0.2476	0.0830	2.98	.004
	Parity	0.0111	0.0747	0.15	.882
	ICV	0.6826	0.1147	5.95	<.001
	Age at scan	-0.3190	0.1121	–2.85	.005
	Intervention group	-0.0410	0.1507	–0.27	.786
Left Amygdala	Partial eta PSS intercept	0.1699	0.0834	2.04	.044
	Partial eta PSS slope	0.0491	0.0837	0.59	.559
	Sex	0.0756	0.0849	0.89	.375
	Parity	-0.0300	0.0764	–0.39	.696
	ICV	0.7185	0.1174	6.12	<.001
	Age at scan	-0.1618	0.1148	–1.41	.162
	Intervention group	-0.0801	0.1543	–0.52	.605

Maternal Perceived Stress Scale (PSS) intercept and slope across pregnancy were first estimated using hierarchical linear modeling. The resulting estimates were then entered as predictors in a general linear model of bilateral hippocampal and amygdala volume, covarying for postconceptional age at scan, intracranial volume (ICV), infant sex, and parity. All statistical tests were two-sided. Covariates and outcomes are standardized in all models.

Supplementary Table 9.

Maternal IPT vs. EUC group by infant sex interaction on hippocampal and amygdala volume.

Brain Region	Predictor	*F*(102)	*p-value*
Right Hippocampus	Parity	0.90	.345
	Sex	0.99	.322
	Age at scan	1.03	.312
	ICV	58.48	<.001
	Intervention group	9.73	.002
	Sex*Intervention group	0.04	.837
Left Hippocampus	Parity	0.00	.976
	Sex	0.02	.880
	Age at scan	0.34	.559
	ICV	23.95	<.001
	Intervention group	1.89	.173
	Sex*Intervention group	0.17	.679
Right Amygdala	Parity	0.00	.950
	Sex	10.51	.002
	Age at scan	6.59	.012
	ICV	34.32	<.001
	Intervention group	0.06	.810
	Sex*Intervention group	0.23	.633
Left Amygdala	Parity	0.24	.628
	Sex	2.22	.139
	Age at scan	0.77	.383
	ICV	32.85	<.001
	Intervention group	0.47	.495
	Sex*Intervention group	0.02	.897

Brain Region	Predictor	Estimate (Δ)	SE	t(102)	*p-value*
Right Hippocampus	Intervention group (Female)	-60.05	27.79	-2.16	.033
	Intervention group (Male)	-68.35	29.77	-2.30	.024
Left Hippocampus	Intervention group (Female)	-43.50	33.02	-1.32	.191
	Intervention group (Male)	-23.65	35.38	-0.67	.505
Right Amygdala	Intervention group (Female)	1.15	6.82	0.17	.866
	Intervention group (Male)	-3.59	7.31	-0.49	.624
Left Amygdala	Intervention group (Female)	-3.71	6.18	-0.60	.549
	Intervention group (Male)	-2.55	6.62	-0.39	.701

A general linear model was used to test the interaction between maternal randomization group and infant sex on bilateral hippocampal and amygdala volume, covarying for postconceptional age at scan, intracranial volume (ICV), and parity. To account for multiple comparisons across the four subcortical regions (bilateral

hippocampus and amygdala), a Bonferroni correction was applied (corrected $\alpha = 0.0125$), and all statistical tests were two-sided. The upper panel reports omnibus tests of fixed effects, including the Sex × Intervention Group interaction. The lower panel reports simple effects of intervention group estimated separately within female and male infants. Estimates (Δ) are expressed as adjusted differences in volume (mm^3) relative to the reference group, enhanced usual care (EUC).

Supplementary Figure 1.

Timeline of the parent randomized clinical trial and observational infant follow-up.

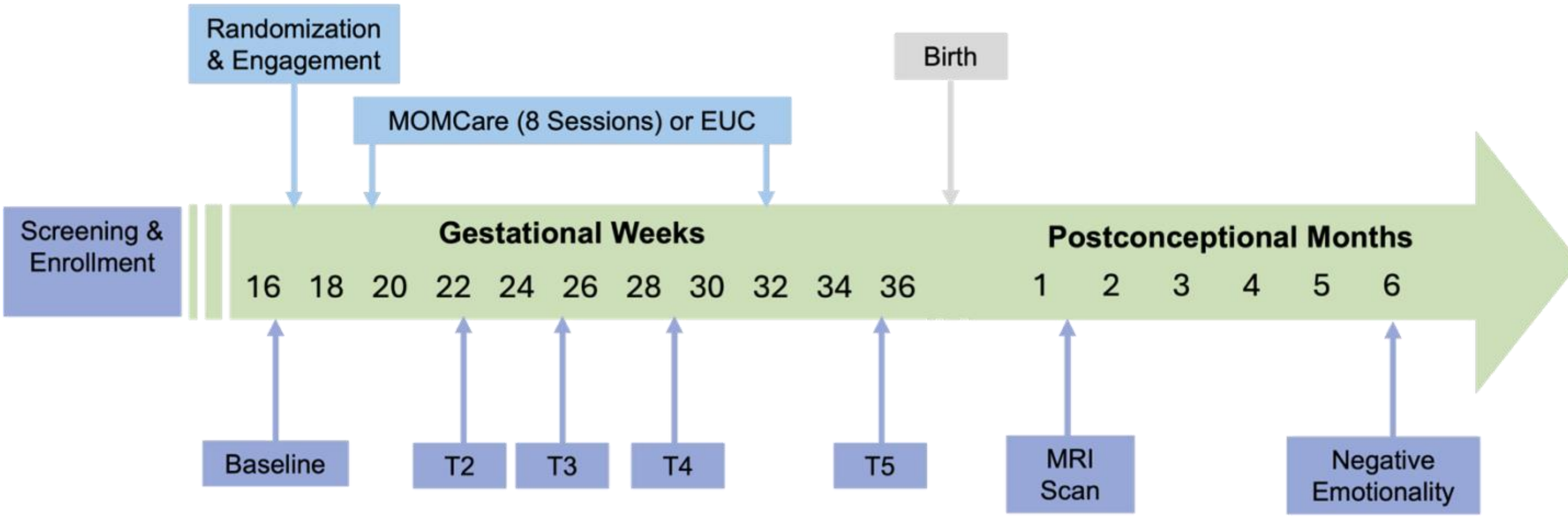

Participants were randomized to brief interpersonal psychotherapy (IPT) or enhanced usual care (EUC) during pregnancy. Maternal assessments were conducted from baseline through late gestation, followed by infant structural MRI at approximately 6 weeks of age and behavioral assessment of negative emotionality at 6 months.

Supplementary Figure 2

Hippocampal and amygdala volume in infants of mothers randomized to IPT vs. EUC, unadjusted for covariates (raw values).

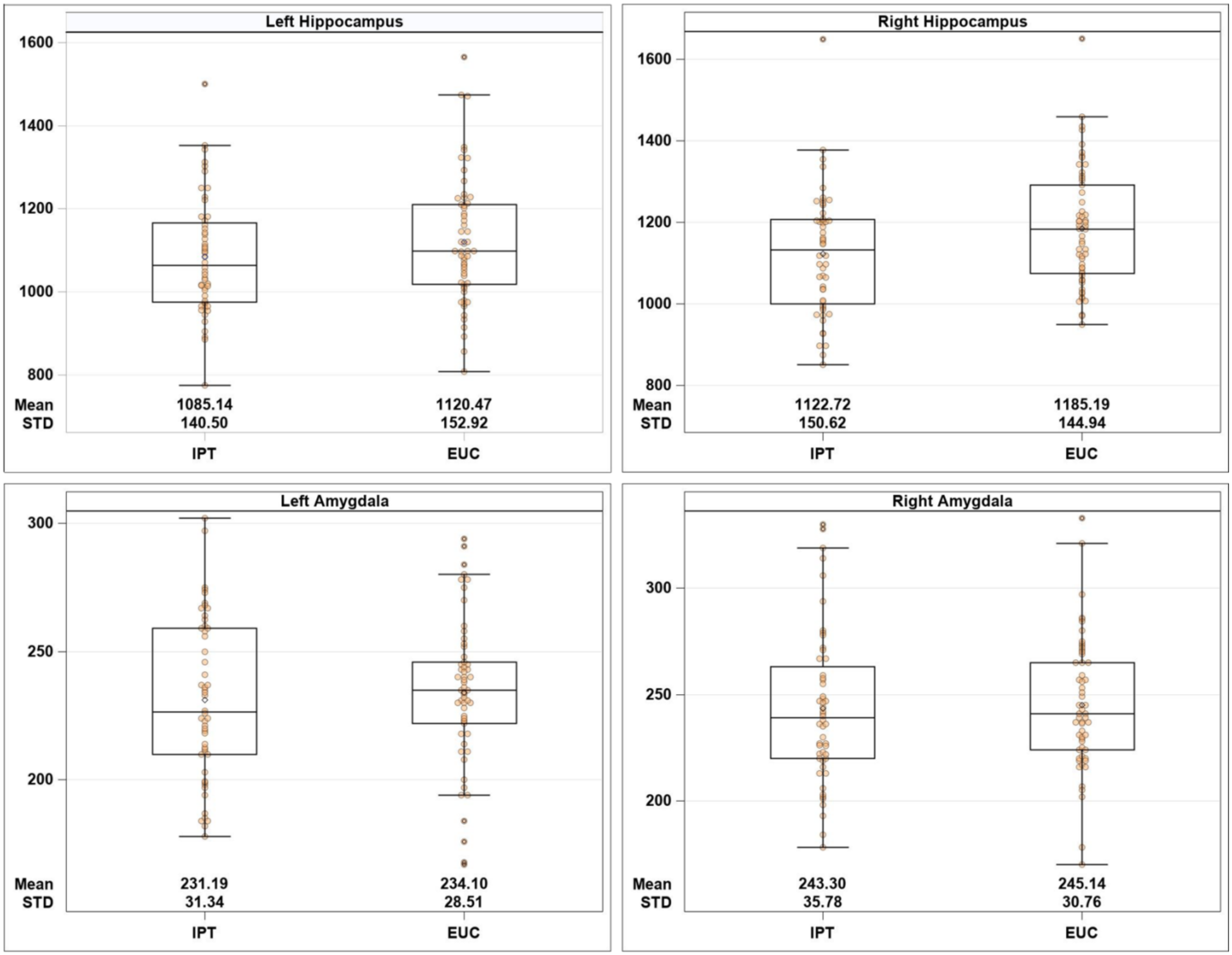

Raw volumetric values (unadjusted for covariates) are plotted. Boxes represent the interquartile range (IQR), center lines indicate the median, whiskers extend to the minimum and maximum within 1.5 × IQR, and circles represent individual data points.
