# Supplementary material for: Infant hippocampal and amygdala volume in an observational follow-up from a randomized clinical trial for maternal prenatal depression
Source: Nat Commun. 2026 Oct 16;17:10649. doi: 10.1038/s41467-026-78276-4 (PMC13675242; doi:10.1038/s41467-026-78276-4)
Supplement: Supplementary file 2 [file 41467_2026_78276_MOESM2_ESM.pdf]

nature portfolio

Corresponding author(s): Elysia Davis

Last updated by author(s): 7/20/2026

Reporting Summary

Nature Portfolio wishes to improve the reproducibility of the work that we publish. This form provides structure for consistency and transparency in reporting. For further information on Nature Portfolio policies, see our Editorial Policies and the Editorial Policy Checklist.

Statistics

For all statistical analyses, confirm that the following items are present in the figure legend, table legend, main text, or Methods section.

n/a	Confirmed	
☐	☒	The exact sample size (n) for each experimental group/condition, given as a discrete number and unit of measurement
☐	☒	A statement on whether measurements were taken from distinct samples or whether the same sample was measured repeatedly
☐	☒	The statistical test(s) used AND whether they are one- or two-sided<br>*Only common tests should be described solely by name; describe more complex techniques in the Methods section.*
☐	☒	A description of all covariates tested
☐	☒	A description of any assumptions or corrections, such as tests of normality and adjustment for multiple comparisons
☐	☒	A full description of the statistical parameters including central tendency (e.g. means) or other basic estimates (e.g. regression coefficient) AND variation (e.g. standard deviation) or associated estimates of uncertainty (e.g. confidence intervals)
☐	☒	For null hypothesis testing, the test statistic (e.g. F, t, r) with confidence intervals, effect sizes, degrees of freedom and P value noted<br>*Give P values as exact values whenever suitable.*
☒	☐	For Bayesian analysis, information on the choice of priors and Markov chain Monte Carlo settings
☐	☒	For hierarchical and complex designs, identification of the appropriate level for tests and full reporting of outcomes
☐	☒	Estimates of effect sizes (e.g. Cohen's d, Pearson's r), indicating how they were calculated

Our web collection on statistics for biologists contains articles on many of the points above.

Software and code

Policy information about availability of computer code

Data collection	Participant questionnaire data were collected and managed using REDCap (Research Electronic Data Capture). Clinical and obstetric data were obtained through medical record review, and MRI data were acquired on a Siemens Skyra 3T MRI system.
Data analysis	All analyses were conducted in SAS version 9.4.

For manuscripts utilizing custom algorithms or software that are central to the research but not yet described in published literature, software must be made available to editors and reviewers. We strongly encourage code deposition in a community repository (e.g. GitHub). See the Nature Portfolio guidelines for submitting code & software for further information.

Data

Policy information about availability of data

All manuscripts must include a data availability statement. This statement should provide the following information, where applicable:

- Accession codes, unique identifiers, or web links for publicly available datasets
- A description of any restrictions on data availability
- For clinical datasets or third party data, please ensure that the statement adheres to our policy

The behavioral and imaging data generated in this study have been deposited in the University of Denver Digital Commons database under accession code https://doi.org/10.56902/Data.Psych.2026.1.

Research involving human participants, their data, or biological material

Policy information about studies with human participants or human data. See also policy information about sex, gender (identity/presentation), and sexual orientation and race, ethnicity and racism.

Reporting on sex and gender	Infant biological sex was obtained from medical records; 27 (51.9%) infants were female in the IPT group and 31 (54.4%) were female in the EUC group. Infant sex (male/female) was included as a covariate in all statistical models. We also conducted exploratory analyses of sex-by-intervention group interactions.
Reporting on race, ethnicity, or other socially relevant groupings	Research staff blinded to the condition collected participant self-reported sociodemographic characteristics, including birthdate, socioeconomic status, cohabitation with a partner, marital status, educational attainment, and race and ethnicity. Income-to-needs ratio (INR) was calculated by dividing the total reported household income for the past year by the federal poverty threshold for that year corresponding to the number of persons living in the household specified by the U.S. Census Bureau.
Population characteristics	See above.
Recruitment	Participants were recruited from obstetric clinics at two major medical centers in the Denver, Colorado area. Initial screening was performed based on medical record review. Eligibility criteria included 18 to 45 years of age, English speaking, 25 weeks' gestational age (GA) or less, singleton pregnancy, and endorsing elevated depression symptoms when screened as part of standard of care (Edinburgh Postnatal Depression Scale [EPDS] score ≥10). Participants were compensated for completing study assessments, including the prenatal questionnaires, the infant MRI visit, and the 6-month infant behavioral assessment. Because enrollment required elevated depressive symptoms and willingness to participate, individuals who declined participation or who did not attend obstetric care are not represented. Further, enrollment was limited to English-speaking individuals within a single metropolitan area (Denver, Colorado). These factors may limit the generalizability of study findings.
Ethics oversight	The Colorado Multiple Institutional Review Board (COMIRB) and the University of Denver Institutional Review Boards for the Protection of Human Subjects approved study protocols.

Note that full information on the approval of the study protocol must also be provided in the manuscript.

Field-specific reporting

Please select the one below that is the best fit for your research. If you are not sure, read the appropriate sections before making your selection.

☐ Life sciences ☒ Behavioural & social sciences ☐ Ecological, evolutionary & environmental sciences

For a reference copy of the document with all sections, see nature.com/documents/nr-reporting-summary-flat.pdf

Behavioural & social sciences study design

All studies must disclose on these points even when the disclosure is negative.

Study description	This study is an observational follow-up of a randomized clinical trial (RCT), employing a quantitative design with maternal mental health assessed repeatedly across pregnancy through standardized questionnaires, infant brain development assessed via structural MRI and infant negative emotionality assessed via behavioral observation at 6 months.
Research sample	The research sample comprised 109 infants and their mothers, drawn from participants in the Care Project, a randomized clinical trial of a prenatal depression intervention (NCT03011801). Mothers were on average 29.96 years of age and 18 weeks' gestational age at recruitment; 58 infants (53.2%) were female. The sample was racially, ethnically, and socioeconomically diverse: 49.5% non-Hispanic/ Latine White, 18.3% Latine, 22.9% multiracial/multiethnic, 7.3% Black, and 1.8% Asian, with 33.9% living at or near the federal poverty threshold (below 200% household income-to-needs ratio). Mothers were recruited through routine obstetric depression screening and were enrolled on the basis of elevated depressive symptoms (Edinburgh Postnatal Depression Scale ≥10); the sample is therefore representative of pregnant individuals with elevated prenatal depresssion receiving obstetric care in a U.S. urban setting rather than of the general pregnant population. This sample was chosen because the parent trial's randomized design allows us to conduct an observational follow up study to examine structural brain development in infants of mothers assigned to a prenatal depression intervention and those assigned to a comparison condition.
Sampling strategy	Post hoc power analyses conducted using the pwr package in R indicated >85% power to detect an effect size of d=0.6.
Data collection	Participants completed self-report depression measures, The Edinburgh Postnatal Depression Scale (EPDS) and the Depression Symptom Checklist (SCL-20) in REDCap three to five times across pregnancy: 16.41 (4.08) weeks (prior to intervention), followed by subsequent assessments at 22.10 (4.01) weeks, 25.61 (4.08) weeks, 29.42 (4.18) weeks (near the end of active IPT), and 35.73 (1.58) weeks (end of gestation). The EPDS was administered at 6 weeks postpartum, concurrent with the MRI visit. In addition to measures of depression, participants also completed the Spielberger State-Trait Anxiety Inventory (STAI) and the Perceived Stress Scale (PSS). Infants were scanned during natural sleep. A Siemens Skyra 3T MRI system equipped with a 20-channel head coil at the Brain Imaging Center at the University of Colorado Anschutz Medical Campus was used. Behavioral assessment of infant negative emotionality was assessed using the Robot Behavioral Task from the Laboratory Temperament Assessment Battery (Lab-TAB). All maternal and infant assessments were performed by evaluators blinded to condition. Gestational age (GA) and postconceptional age at scan (PCA) were calculated from medical records review, applying American College of Obstetricians and Gynecologists (ACOG) guidelines of first-

	trimester ultrasound and/or date of the last menstrual period. Postconceptional age at scan was calculated as the sum of gestational age at birth and the infant's chronological age since birth (at the time of the MRI scan). Additional obstetric information, including parity and obstetric complications, maternal height and weight, and infant sex and birthweight, was obtained from medical records. Staff were not formally blinded to the study hypotheses; however, neonatal images were segmented by staff masked to group assignment, with volumetric outcomes derived through standardized, automated pipelines, and infant behavioral responses were coded by staff masked to group assignment.
Timing	Recruitment began July 2017 and ended August 2021 with a pause from mid-April through mid-June due the COVID-10 pandemic.
Data exclusions	Seven individuals were excluded prior to the MRI scan for the following reasons: twin pregnancy (n=1), therapeutic/spontaneous abortion (n=3), still born (n=1), MRI contraindication (n=1) and major fetal chromosomal abnormality (n=1). After MRI scan, data were excluded for the following: not falling asleep/waking up mid scan = 4 and failing image QC (n=3).
Non-participation	Within the RCT, 115 were randomly assigned to IPT and 119 to EUC. Within the IPT group 56 were lost to follow up (declined MRI =14, missed due to other reasons =42, excluded (n=3). Within the EUC group 55 were lost to follow up (declined MRI = 19, missed due to other reasons = 36, excluded =4). See Figure 1 Consort diagram for further details.
Randomization	Eligible participants were assessed at baseline for depression and then randomly assigned via parallel assignment to Interpersonal Psychotherapy (IPT) or Enhanced Usual Care (EUC) using a computer-generated random numbers sequence. Participants were randomized in blocks of two and stratified on current Major Depressive Disorder (MDD), gestational age (GA) (above or below 15 weeks), and Medicaid status for each study arm.

Reporting for specific materials, systems and methods

We require information from authors about some types of materials, experimental systems and methods used in many studies. Here, indicate whether each material, system or method listed is relevant to your study. If you are not sure if a list item applies to your research, read the appropriate section before selecting a response.

Materials & experimental systems

n/a	Involved in the study
☒	☐ Antibodies
☒	☐ Eukaryotic cell lines
☒	☐ Palaeontology and archaeology
☒	☐ Animals and other organisms
☒	☐ Clinical data
☒	☐ Dual use research of concern
☒	☐ Plants

Methods

n/a	Involved in the study
☒	☐ ChIP-seq
☒	☐ Flow cytometry
☐	☒ MRI-based neuroimaging

Plants

Seed stocks	n/a
Novel plant genotypes	n/a
Authentication	n/a

Magnetic resonance imaging

Experimental design

Design type	n/a
Design specifications	n/a
Behavioral performance measures	n/a

Acquisition

Imaging type(s)
Structural MRI

Field strength
A Siemens Skyra 3T MRI system equipped with a 20-channel head coil at the Brain Imaging Center at the University of Colorado Anschutz Medical Campus was used.

Sequence & imaging parameters
T1-weighted (T1w) images were obtained using a three-dimensional magnetization-prepared rapid gradient echo sequence (repetition time = 1900 ms; echo time = 3.07 ms; inversion time = 900 ms; flip angle 9°; 4 min 26 s) and T2-weighted (T2w) images were obtained with a 3D fast turbo spin echo sequence (repetition time 3200 ms; echo time = 408 ms; flip angle var; 4 min 43 s). The spatial resolution was a 0.82 × 0.82 × 0.8 mm voxel for T1w and 0.86 mm × 0.86 mm × 0.8 mm voxel for T2w.

Area of acquisition
Whole brain

Diffusion MRI ☐ Used ☒ Not used

Preprocessing

Preprocessing software
The T1w and T2w brain images were corrected for intensity non-uniformity via N4 60, rigidly transformed to a prior pediatric neonate atlas in stereotaxic space. Brain masking was performed via the 3D UNet-based brain masking tool in ANTSPyNet using both T1w and T2w images jointly, including also extra-axial cerebro-spinal fluid spaces in the brain mask. All brain masks were corrected manually in ITK-SNAP.

Normalization
The T1w and T2w brain images were corrected for intensity non-uniformity via N4, rigidly transformed to a prior pediatric neonate atlas in stereotaxic space.

Normalization template
As above, rigid transformation to a prior pediatric neonate atlas in stereotaxic space (61).

Noise and artifact removal
Image quality control (QC) was assessed using a four-point scale (0–3) 58 adapted in-house for infant scanning. The criteria for exclusion was a QC score of 0, indicating artifact contamination, rendering the image processing unreliable mainly due to subject motion. Three scans were excluded based on failed T1w image quality. For subjects who had artifact contamination in T2w image, T2 data were imputed from a passing T1w image (n=10).

Volume censoring
n/a

Statistical modeling & inference

Model type and settings
General linear models were used to test group differences in infant subcortical brain volumes. Partial correlations were run to test associations between left and right amygdala and hippocampal volume and infant negative emotionality.

Effect(s) tested
Primary analyses examined whether infant bilateral amygdala and hippocampal volumes differed between infants of mothers randomized to IPT vs. EUC. Secondary analyses examined associations between neonatal volume and infant negative emotionality at 6 months. Also examined whether depression, anxiety or perceived stress symptom slopes were associated with infant hippocampal or amygdala volume.

Specify type of analysis: ☐ Whole brain ☒ ROI-based ☐ Both

Anatomical location(s)
Hippocampal and amygdala regions were defined as in (see ref 64).

Statistic type for inference
(See Eklund et al. 2016)
n/a

Correction
n/a

Models & analysis

n/a	Involved in the study
☒	☐ Functional and/or effective connectivity
☒	☐ Graph analysis
☒	☐ Multivariate modeling or predictive analysis
