# Supplementary material for: Infant hippocampal and amygdala volume in an observational follow-up from a randomized clinical trial for maternal prenatal depression
Source: Nat Commun. 2026 Oct 16;17:10649. doi: 10.1038/s41467-026-78276-4 (PMC13675242; doi:10.1038/s41467-026-78276-4)
Supplement: Supplementary file 3 [file 41467_2026_78276_MOESM3_ESM.pdf]

nature portfolio

Peer Review File

Infant hippocampal and amygdala volume in an observational follow-up from a randomized clinical trial for maternal prenatal depression

Corresponding Author: Professor Elysia Davis

This file contains all reviewer reports in order by version, followed by all author rebuttals in order by version.

Version 0:

Reviewer comments:

Reviewer #1

(Remarks to the Author)
This is an important paper describing novel results leveraging a RCT to treat perinatal depression as a means to gain experimental control in DOHAD research. The scientific rationale is well argued. The design is well considered and methods sound. The discussion is thorough and interesting in its consideration of the results in relation to existing research Pls see below for some specific issues to consider addressing.

1. Errors in formatting citations
2. Suggest results of MOMCare should be moved out of procedures section and into background
3. Line 153 is reporting the reduction in rates TO (?), so that the %s given are the %s at the end of the intervention trial? Pls clarify
4. Overall flow of data collection is not described; gestational ages participants entered study, age of when infants underwent MRI scan, frequency mood assessed, etc
5. Line 224 Medicaid status; suggest re writing to say insurance status and specify options (and in Table 1)
6. Is gestational age at birth controlled for in analyses by the inclusion of gestational age at scan? Clarifying the procedure, e.g., at what age were babies scanned, would help clarify this
7. Medication is discussed in the authors' prior report on the IPT intervention, but not here. What % of participants were taking psychotropic medications, and were there differences between the groups? Shouldn't medication use in pregnancy be controlled for in analyses?
8. Line 242, no significant differences. If results are not significant by a p test, pls describe as not significant.
9. Line 289: discussion of mechanisms for transmission of maternal mood to fetus, affecting development, is useful. However, this discussion raises the question as to whether the word 'how' and the associated framing of the paper's focus in line 70 is the best possible framing. This paper really isn't looking at 'how', it isn't looking at mechanism of transmission. Also, though this is a good discussion, might this 'space' be better used for more detailed discussion relating imaging results to other studies? That is, discussing in more detail results of this study in relation to similar studies on brain outcomes in relation to prenatal exposure to maternal depression, anxiety, etc?
10. Line 293 missing a parenthesis
11. In table 1, is postconceptual age the age at the scanning session?
12. Line 324, is correlate the appropriate word here as this study is highlighting the strengths of more rigorously conducted studies.

Reviewer #2

(Remarks to the Author)
The paper at hand describes a very interesting RCT, designed to shed light on the influence of maternal depression on offspring brain development. The study produced highly valuable data, however, the analysis and presentation of results is slim and limits the current value for the community.
The authors decided to restrict their analysis to two regions of interest; the hippocampus and the amygdala. The decision appears to rely on previous work linking maternal depression to the limbic system. At least a clearer justification for omitting

an analysis of other imaging derived measures would have been warranted. Furthermore, among the two regions of interest, only the results for the hippocampus are visualized. I believe amygdala results are not shown due to non-significant findings; however, this data would help the reader put the strength of hippocampus findings into context.
The authors include age, intracranial volume, infant sex and parity as covariates. Covariate statistics are not shown. It would have been interesting to know if these are associated with volume beyond the treatment effects. This appears particularly relevant for age considering their 'age acceleration' interpretation. Further, analyses comparing treatment effects for each infant sex separately would additionally help given known sex differences in brain development. Finally, the degree to which image quality may affect the results is not investigated as this is not a covariate in the analysis.
The authors make a strong case that their RCT overcomes limitations of correlational designs. This is correct when it comes to their statistical analysis (the group effect), however, it does not apply to their interpretation of results. Specifically, the authors interpret the negative sign in the treatment effect as an indication of accelerated aging in the EUC group. This interpretation is correlational in nature as the authors have neither longitudinal data to quantify trajectories, nor (normative) control data that confirms that the effect resides in the EUC and not in the IPT group. The authors may want to consider toning down their conclusions in line 274/275 ('likely reflects mitigation of this accelerated process'), or at least discuss that this part of the study is indirect evidence of correlational nature. It is noted briefly in lines 312 onward but I would consider emphasizing this more.
To conclude, while I found the data set created in the study highly interesting and believe that the study can make a very valuable contribution to the field, I would suggest the authors re-evaluate if there are more analyses / results that they could provide the readers with, or if at least the data could be shared openly so that others could support in exploring this valuable data set.

Reviewer #3

(Remarks to the Author)
This study reports on the impact of a maternal prenatal depression intervention (brief interpersonal psychotherapy) on infant amygdala and hippocampus volumes. The research question investigated is important, and findings have important implications for preventative interventions for children's health and wellbeing. The manuscript was also well written. I reviewed this work together with one of my doctoral students, and our amalgamated comments are below.

Major points
1. It is a hypothesis that reduced depressive symptoms led to hippocampal volume change. However, currently this is not tested. Were hippocampal volumes correlated with depressive symptom change? Were there other symptoms (e.g., anxiety symptoms) or measures of stress assessed before and after the intervention?
2. While scans were performed soon after birth (~6 weeks), it is still possible that the early postnatal environment could contribute. Could the authors examine the effect of IPT on postnatal maternal depression, and the effect of postnatal maternal depression on infant brain structure? This would help to better disentangle the effects of prenatal vs postnatal maternal psychopathology on infant outcomes, and biological vs environmental influences on brain development.
3. In the analysis, covariates included postconceptional age at scan, intracranial volume, infant sex, and parity. I wonder if the authors have considered other confounding factors such as maternal physical health, obstetric complications, infant birthweight? Further, were QC scores correlated with subcortical volumes? The authors may wish to include these scores as covariates to increase specificity in the outcome variable. Related, were there any multicollinearity issues with both sex and intracranial volume being included in the model together?
4. Given prior research has found sex differences in the association between maternal prenatal depression and subcortical volumes, did the authors consider looking at sex differences in their own analyses?
5. Please provide a table outlining statistics four full models including effects of covariates. It would be of interest to know if intracranial volume differed between groups.
6. Were there any statistical outliers and were residuals from models normally distributed?
7. Are the results adjusted for multiple comparisons? If not, please provide justification.
8. Have the authors collected any infant behavioural and socioemotional data? It would be interesting to examine whether brain structure may mediate the effect of IPT intervention on infant outcomes.

Minor points
1. Incorrect in-text referencing on page 1
2. Measures: (1) It would be helpful to include internal consistency; (2) the timepoints when the measures were administered are not clear
3. Does figure 3 depict raw hippocampal volumes or volumes with covariates regressed out?
4. On page 4, the authors state "While such accelerated maturation …these changes may increase the lifetime risk of mental illness and, in fact, have been associated with behavioral outcomes such as increased negative emotionality and cognitive impairments 22,23" These studies looked at white matter and not subcortical volume, so relevance is unclear.

Version 1:

Reviewer comments:

Reviewer #1

(Remarks to the Author)

The authors very effectively responded to the reviewers' feedback, including the excellent comments made by other reviewers. This is a clearly-written paper reporting interesting, novel, and important results.
However, I am a bit troubled by the lack of association between change in maternal mood and infant hippocampal volume, esp in the context of 1 out of 4 a priori brain hypotheses being supported, albeit there now is correction for multiple comparisons.
In addition to the interpretations offered, perhaps the authors should consider: failure of randomization that is not detected with current subject phenotyping; the role of social support experienced via IPT (perhaps this even could be tested?).
Finally, I do have a question about the group comparison in Table 1 that maybe could account for the lack of effect of mood improvement in relation to infant MRI outcomes: the only statistically significant difference between groups is parity/being nulliparous; however, when you look at the % in the rows of demographic variables, there are hints of differences: nearly 2x the number of Hispanic/Latine in the EUC group; > Blacks and Asians in the EUC group; higher % White in the IPT group; and then > percent experiencing poverty in the IPT group (could this be triple checked? In the USA, as we all know, people of color tend to experience higher rates of poverty yet the IPT group with a higher % of White participants has a higher % of people experiencing poverty). Putting demographics and parity together, to the extent that having more children is stressful, and being a person of color in the USA is stressful, it would seem the EUC group is overall experiencing more stressors/life stress. Are these 'soft markers' of group differences that may not be affected by IPT yet relate to infant hippocampal volume? Independent of group assignment, are these variables related to infant hippocampal volume?

Reviewer #2

(Remarks to the Author)
The authors have addressed all my points.

Reviewer #3

(Remarks to the Author)
We thank the authors for their responses to our comments, and for their comprehensive revision. We believe the manuscript is acceptable for publication in its current form, and will make an excellent contribution to the literature.

Reviewer #4

(Remarks to the Author)
I co-reviewed this manuscript with one of the reviewers who provided the listed reports. This is part of the Nature Communications initiative to facilitate training in peer review and to provide appropriate recognition for Early Career Researchers who co-review manuscripts.

Version 2:

Reviewer comments:

Reviewer #1

(Remarks to the Author)
Authors have addressed my additional questions.

Open Access This Peer Review File is licensed under a Creative Commons Attribution 4.0 International License, which permits use, sharing, adaptation, distribution and reproduction in any medium or format, as long as you give appropriate credit to the original author(s) and the source, provide a link to the Creative Commons license, and indicate if changes were made.
In cases where reviewers are anonymous, credit should be given to 'Anonymous Referee' and the source.
The images or other third party material in this Peer Review File are included in the article's Creative Commons license, unless indicated otherwise in a credit line to the material. If material is not included in the article's Creative Commons license and your intended use is not permitted by statutory regulation or exceeds the permitted use, you will need to obtain permission directly from the copyright holder.
To view a copy of this license, visit https://creativecommons.org/licenses/by/4.0/

Summary

Reviewer #1 (Remarks to the Author):

This is an important paper describing novel results leveraging a RCT to treat perinatal depression as a means to gain experimental control in DOHAD research. The scientific rationale is well argued. The design is well considered and methods sound. The discussion is thorough and interesting in its consideration of the results in relation to existing research Pls see below for some specific issues to consider addressing.

1. Errors in formatting citations.

Thank you, these citation formatting errors have been fixed.

2. Suggest that the results of MOMCare should be moved out of the procedures section and into the background.

We have now moved the results of MOMCare from the procedures section into the background as follows:

Pgs. 4-5. "Results from our previously published findings have established that a brief, noninvasive psychosocial intervention (brief interpersonal psychotherapy [IPT]) rapidly and substantially reduces maternal prenatal depression before birth. Specifically, the RCT examined depression symptoms across pregnancy among 234 individuals randomized to either brief IPT or enhanced usual care (EUC). Significant group differences were observed such that the IPT group displayed a significantly greater decline in both symptoms and diagnosis as compared to the IPT group. Specifically, individuals in the IPT group showed a 47% decrease on the Symptom Checklist (SCL-20) and a 52.5% decrease on the Edinburgh Postnatal Depression Scale (EPDS) from baseline to the end of gestation; in contrast, those in the enhanced usual care (EUC) condition showed a 13.0% (SCL-20) and 32.3% (EPDS) decrease in symptoms *(d = 0.40; 95%CI, 0.06-0.74*. Further, participants assigned to IPT showed significantly greater MDD remittance relative to EUC. MDD rate from baseline to the end of gestation decreased 5-fold from 36.5% to 6.1% for IPT participants, compared to a smaller decrease (37 to 26.1%) for EUC (odds ratio, 4.99; 95% CI, 2.08-11.97)."

3. Line 153 is reporting the reduction in rates TO (?), so that the %s given are the %s at the end of the intervention trial? Pls clarify.

Yes, the reviewer is correct. We have now clarified that the percentages are the rates of MDD at the beginning and end of the trial for each group.

Pg. 5. "Further, participants assigned to IPT showed significantly greater MDD remittance relative to EUC. MDD rate from baseline to the end of gestation decreased 5-fold from 36.5% to 6.1% for IPT participants, compared to a smaller decrease (37 to 26.1%) for EUC (odds ratio, 4.99; 95% CI, 2.08-11.97)."

4. Overall flow of data collection is not described; gestational ages participants entered study, age of when infants underwent MRI scan, frequency mood assessed, etc

We thank the reviewer for their comment and have added additional information to improve our description of the overall flow of data collection as follows across the methods and results

section, including the timing of participant recruitment, gestational age, frequency of depression assessment, and age at the time of infant MRI scan. Such details have been incorporated into the text (see below for examples of additions to the methods), and the reviewer may also refer to Table 1 and Supplemental Figure 1 for a new visual depiction of the RCT study timeline of data collection.

Pg. 6. “Participants completed interviews and questionnaires across pregnancy to assess prenatal depressive symptoms. Neonatal amygdala and hippocampal volume were assessed via structural magnetic resonance imaging (MRI) during natural sleep at 44.8 weeks’ postconceptional age (~ 6 weeks old) on average. Infant negative emotionality was assessed via behavioral observation at 6 months (see Supplementary Figure 1 for RCT timeline).”

Pg. 12. “Participants were 29.96 years of age and 18 weeks’ gestational age on average at recruitment.

Pg. 7. “Participants completed self-report depression measures via REDCap three to five times across pregnancy: 16.41 (4.08) weeks (prior to intervention), followed by subsequent assessments at: 22.10 (4.01) weeks, 25.61 (4.08) weeks, 29.42 (4.18) weeks (near the end of active IPT), and 35.73 (1.58) weeks (end of gestation). The EPDS was administered at 6 weeks postpartum, concurrent with the MRI visit; see Figure 2.”

Pg. 6. “Neonatal amygdala and hippocampal volume were assessed via structural magnetic resonance imaging (MRI) during natural sleep at 44.8 weeks’ postconceptional age (~ 6 weeks old) on average.”

5. Line 224 Medicaid status; suggest re writing to say insurance status and specify options (and in Table 1)

In our sample, just over half of the participants were enrolled in Medicaid, while the remainder had a mix of other insurance types. Because Medicaid was the predominant form of coverage and we did not systematically collect information on other specific insurance options, we reported insurance status as Medicaid enrollment (yes/no).

6. Is gestational age at birth controlled for in analyses by the inclusion of gestational age at scan? Clarifying the procedure, e.g., at what age were babies scanned, would help clarify this.

We thank the reviewer for the opportunity to clarify our procedure. We clarify that postconceptional age at scan was calculated as the sum of gestational age at birth and the infant’s chronological age since birth at the time of the MRI scan. This approach is used to fully account for chronological age from conception to the time of the MRI scan, consistent with the neonatal MRI literature (e.g., PMID: 32912873; PMID: 35945202). This variable, therefore, includes gestational age at birth and was used as a covariate in all primary analyses.

Pg. 9. “Postconceptional age at scan was calculated as the sum of gestational age at birth and the infant’s chronological age since birth (at the time of the MRI scan).”

7. Medication is discussed in the authors’ prior report on the IPT intervention, but not here. What % of participants were taking psychotropic medications, and were there differences between the groups? Shouldn’t medication use in pregnancy be controlled for in analyses?

Based on the reviewers' feedback, we now include a description of the percentage of participants who were taking psychotropic medications in the results and in Table 1. There was no significant difference between groups in rate of psychotropic medication use (25.5% IPT, 21.8% EUC; (χ^2 (1)=0.19, p=0.66).

Secondly, we ran our primary models with psychotropic medication use as an additional covariate. The treatment group difference in right hippocampal volume remained significant ($F(1, 95) = 7.71$, $p = .0066$), with IPT infants showing lower volume relative to EUC (Estimate = -59.19 mm^3, SE = 21.31, 95% CI [-101.50, -16.87]). Psychotropic medication use itself was not a significant predictor of right hippocampal volume ($F(1, 95) = 0.70$, $p = .4046$).

We have now included models with psychotropic medication use as an additional covariate in the supplement (see **Supplementary Table 5**) and reference these in the main text.

8. Line 242, no significant differences. If results are not significant by a p test, pls describe as not significant.

As requested, we have edited the following description of results from "yielded non-significant differences" to "were not statistically significant".

Pg. 10. "Intervention comparisons for the left hippocampus and bilateral amygdala volumes were not statistically significant; left hippocampus: t(103)=-1.41, p=0.16, d=0.28, 95% CI [-0.10 - +0.66]; right amygdala: t(103)=-0.21, p=0.83, d=0.04, 95% CI [-0.34 - +0.42]; and left amygdala: t(103)=-0.70, p=0.49, d=0.11, 95% CI [-0.24 - +0.52])."

9. Line 289: discussion of mechanisms for transmission of maternal mood to fetus, affecting development, is useful. However, this discussion raises the question as to whether the word 'how' and the associated framing of the paper's focus in line 70 is the best possible framing. This paper really isn't looking at 'how', it isn't looking at mechanism of transmission. Also, though this is a good discussion, might this 'space' be better used for more detailed discussion relating imaging results to other studies? That is, discussing in more detail results of this study in relation to similar studies on brain outcomes in relation to prenatal exposure to maternal depression, anxiety, etc?

We appreciate the reviewer's thoughtful feedback regarding the framing of the manuscript. In response, we have revised the text to more accurately reflect the focus of the study. Specifically, we now emphasize that the focus of the paper was to test the impact of implementing an efficacious depression intervention on neonatal brain volume using a randomized clinical trial design. This study applies a more experimental approach to test differences in offspring born to mothers in two treatment groups. The reviewer is correct that this paper does not investigate the underlying mechanisms of transmission.

Pg. 3. "To test whether changes in depressive symptoms across pregnancy were associated with infant amygdala and hippocampal volume, symptom trajectories, assessed via the SCL-20 and EPDS, were entered as separate predictors in the model. In addition to depression, other mental health indicators, including trajectories of maternal anxiety (assessed via the STAI) and stress (assessed via the PSS), were also examined as model predictors. study employed a randomized clinical trial (RCT) to rigorously test the fetal origins hypothesis, aiming to determine whether alleviating maternal depression during pregnancy can yield significant effects on child neurodevelopment [3]. By focusing on the direct impact of prenatal mental health interventions, this research provides the first experimental evidence of how

prenatal maternal mental health influences offspring brain development and identifies potential prevention and intervention strategies that could enhance lifelong health outcomes for future generations."

Secondly, we have expanded our discussion to better contextualize our current experimental findings with findings from correlational studies. We now provide a more detailed discussion of our findings in the context of similar studies examining brain outcomes following prenatal exposure to maternal depression.

Pg. 15-16. "Prior correlational studies in infants link higher prenatal maternal depression to larger hippocampal volume in 2 to 6-week-olds [27] and subcortical brain volume in infants (ages 3-6 months) [26] , supporting the accelerated maturational hypothesis. Further evidence for an accelerated pattern of brain development is observed in a study linking maternal prenatal depression to altered resting state connectivity [74]. However, findings across the infant literature are inconsistent. One study found a sex-specific effect whereby prenatal depression was associated with *smaller* amygdala volumes in males only [28] , while others found null effects with amygdala and hippocampal volume [37–39]. Taken together, this small infant literature does not yield consensus regarding the impact of prenatal maternal mental health on offspring brain development, highlighting the need for experimental studies to clarify these associations."

We also reiterate in the discussion that the biological mechanisms by which maternal depression may influence infant brain development remain untested in the present study.

Pg. 15. "Leveraging the power of an RCT design, these experimental findings show that infants of mothers who received IPT had altered early hippocampal volume compared to infants of mothers in the enhanced usual care group. Specifically, group differences were observed such that infants of mothers who received IPT had significantly smaller right hippocampal volume compared to EUC. To test the functional correlates of enlarged hippocampal volume, we examined links with negative emotionality assessed via behavioral observation at 6 months of age. Findings showed that larger neonatal hippocampal volume was associated with greater subsequent infant negative emotionality. These preliminary data provide evidence that larger hippocampal volume may have implications for emerging emotional development. However, the long-term developmental significance and biological mechanisms underlying these differences are untested."

10. Line 293 missing a parenthesis

We thank the reviewer for catching this typo and have added in the missing parenthesis.

11. In table 1, is postconceptual age the age at the scanning session?

Yes, we have clarified that postconceptional age in Table 1 refers to the age at the scanning session by revising the label to read: *postconceptional age at scan*.

For further clarification, we have also added a definition of postconceptional age in the Methods section, as follows:

Pg 9: "Postconceptional age at scan was calculated as the sum of gestational age at birth and the infant's chronological age since birth at the time of the MRI scan".

12. Line 324, is correlate the appropriate word here as this study is highlighting the strengths of more rigorously conducted studies.

We thank the reviewer for this helpful suggestion. In response, we have revised the sentence to reflect the rigor of the randomized design and experimental approach.

Pg. 18. "Exploring how these early structural changes ***predict*** later behavioral outcomes beyond infancy will provide valuable insights into early identification and intervention strategies that could mitigate mental health risks across generations".

Reviewer #2 (Remarks to the Author):

The paper at hand describes a very interesting RCT, designed to shed light on the influence of maternal depression on offspring brain development. The study produced highly valuable data, however, the analysis and presentation of results is slim and limits the current value for the community.

1. The authors decided to restrict their analysis to two regions of interest; the hippocampus and the amygdala. The decision appears to rely on previous work linking maternal depression to the limbic system. At least a clearer justification for omitting an analysis of other imaging derived measures would have been warranted. Furthermore, among the two regions of interest, only the results for the hippocampus are visualized. I believe amygdala results are not shown due to non-significant findings; however, this data would help the reader put the strength of hippocampus findings into context.

As noted by the reviewer, we focused our analyses on the hippocampus and amygdala based on 1) a strong body of prior correlational research linking prenatal maternal depression to structural alterations in these specific limbic structures, which are central to affective and stress-related processing and 2) experimental animal research indicating these regions are susceptible to prenatal stress exposure. This targeted approach allowed us to test focused *a priori* regions while minimizing the corrections needed when testing across multiple areas. We have added additional evidence and references to further strengthen the rationale for our approach.

Pgs 3-4. "Experimental evidence in rodents [13] and correlational studies in humans suggest that prenatal exposure to maternal depression, a form of early life adversity, has cascading effects on offspring brain development particularly within limbic regions such as the amygdala and the hippocampus (for reviews see [14–16]). The amygdala and hippocampus are two prime candidate regions for investigating the effects of prenatal depression exposure given their critical roles in affective processing, fear learning, and memory [17–20], as well as established links with many psychiatric illnesses, including post-traumatic stress disorder (PTSD) and depression [21–24]. Both regions are highly susceptible to stress and contain high levels of glucocorticoid receptors, increasing their sensitivity to dysregulation of the hypothalamic–pituitary–adrenal (HPA) axis [25]. Cross-species evidence indicates these regions are vulnerable to adversity, including maternal depression, during the prenatal period. In humans, exposure to maternal prenatal depression and distress is associated with volumetric alterations in the amygdala and hippocampus in infancy [26–31] and later childhood and adolescence [32–34]. The vulnerability of these regions is substantiated by experimental rodent and non-human primate research demonstrating that maternal prenatal stress impacts amygdala and hippocampal morphology [13,35]."

Additionally, as requested by the reviewer we now visualize results for the amygdala. **Figure 2** now includes visualizations of both hippocampal and amygdala volumes to provide greater context and allow readers to fully evaluate the pattern of results.

2. The authors include age, intracranial volume, infant sex and parity as covariates. Covariate statistics are not shown. It would have been interesting to know if these are associated with volume beyond the treatment effects. This appears particularly relevant for age considering their 'age acceleration' interpretation.

We have now included a table presenting the full model results with covariate estimates (see **Supplementary Table 2**). Thus, as requested, it is now clear how covariates are associated with volume beyond treatment effects. As expected, intracranial volume (ICV) was significantly associated with both hippocampal and amygdala volume. Other covariates such as infant sex, postconceptional age at scan and parity were not associated with hippocampal or amygdala volume, with the exception of two associations between sex and PCA with right amygdala volume. Limited associations of volume with age at scan likely reflect our study design which constrained the range of postconceptional age at scan (defined as gestational age at birth plus days since birth at scan). This approach minimized developmental or maturational differences in volume due to age.

Finally, the degree to which image quality may affect the results is not investigated as this is not a covariate in the analysis.

We thank the reviewer for raising the issue of image quality. Imaging data were processed under the supervision of co-author Dr. Martin Styner, a leading expert in neonatal neuroimaging, using rigorous, standardized procedures. Image quality control was assessed with an in-house 4-point scale tailored for infant scans. Scans rated 0 (indicating excessive motion) were excluded (n=3). Images underwent standard preprocessing, including bias correction, atlas registration, and multimodal segmentation. Brain masks were manually reviewed and corrected in ITK-SNAP. No scans were excluded due to failed segmentation of the hippocampus or amygdala. These procedures align with current best practices and ensure high-quality data.

In sensitivity analyses, we have now included image quality as a covariate in our primary models. Previously, scans with a score of 0 were removed. We now additionally include QC scores (1–3) as a covariate in all relevant models to account for residual variation in image quality. Image quality was not significantly associated with hippocampal or amygdala volume and all treatment effects remained consistent when adding image quality as an additional covariate to the models (see **Supplementary Table 7** for full model results).

Pg. 13, "Additionally, models were also robust when covarying for imaging quality (see **Supplementary Table 7**).

Further, analyses comparing treatment effects for each infant sex separately would additionally help given known sex differences in brain development.

We completely agree with the reviewer as to the importance of early-life sex differences and appreciate the suggestion to include in this manuscript. Given limited power to detect moderation, we did not include sex-stratified treatment comparisons in our original analyses. We now include these as secondary analyses and report that the intervention group by sex interaction was not statistically significant for hippocampal or amygdala volumes bilaterally.

Pg. 14 “The intervention group by sex interaction was not statistically significant for hippocampal (all ps>.679) or amygdala (all ps >.624) volume (see **Supplementary Table 9** for full models).

A full presentation of these models are included in the supplement and we briefly describe here. For the right hippocampus we show the interaction by sex was non-significant ($F(1, 102) = 0.04$, $p = .837$) and further, follow-up simple slope analyses demonstrated that IPT was associated with significantly lower right hippocampal volume compared to EUC in both females ($\Delta = -60.05$, $p = .033$) and males ($\Delta = -68.35$, $p = .024$), suggesting a consistent treatment effect across sexes. Further we specify that no other significant intervention group by sex interactions were found for the left hippocampus ($F(1, 102) = 0.17$, $p = .679$.) or bilateral amygdala (left: $F(1, 102) = 0.02$, $p = .897$; right: $F(1, 102) = 0.23$, $p = .624$). In all cases, analyses reveal a similar pattern for males (left hippocampus: $\Delta = -23.65$, $p = .501$, and left: $\Delta = -2.5515$, $p = .7009$ and right: $\Delta = -3.5924$, $p = .6242$ amygdala) and females (left hippocampus: $\Delta = -43.5037$, $p = .1906$, and left: $\Delta = -3.7133$, $p = .5493$ and right: $\Delta = 1.1529$, $p = .8662$ amygdala).

3. The authors make a strong case that their RCT overcomes limitations of correlational designs. This is correct when it comes to their statistical analysis (the group effect), however, it does not apply to their interpretation of results. Specifically, the authors interpret the negative sign in the treatment effect as an indication of accelerated aging in the EUC group. This interpretation is correlational in nature as the authors have neither longitudinal data to quantify trajectories, nor (normative) control data that confirms that the effect resides in the EUC and not in the IPT group. The authors may want to consider toning down their conclusions in line 274/275 ('likely reflects mitigation of this accelerated process'), or at least discuss that this part of the study is indirect evidence of correlational nature. It is noted briefly in lines 312 onward but I would consider emphasizing this more.

We appreciate this point. We have clarified our language in the manuscript to be clear that while our randomized clinical trial allows us to identify significant group differences in infant brain volume this does not apply to causality in terms of interpretations about underlying developmental processes. We now state clearly that interpretations, such as whether treatment prevents accelerated maturation across development are hypotheses that need to be tested. We have now clarified that in order to more fully interpret these findings, the field needs normative comparison or longitudinal follow-up data. Future studies must clarify the developmental processes that give rise to these early structural differences.

Pgs. 14-15. “Leveraging the power of an RCT design, these experimental findings show that infants of mothers who received IPT had altered early hippocampal volume compared to infants of mothers in the enhanced usual care group. Specifically, group differences were observed such that infants of mothers who received IPT had significantly smaller right hippocampal volume compared to EUC. To test the functional correlates of enlarged hippocampal volume, we examined links with negative emotionality assessed via behavioral observation at 6 months of age. Findings showed that larger neonatal hippocampal volume was associated with greater subsequent infant negative emotionality. These preliminary data provide evidence that larger hippocampal volume may have implications for emerging emotional development. However, the long-term developmental significance and biological mechanisms underlying these differences are untested”.

To conclude, while I found the data set created in the study highly interesting and believe that the study can make a very valuable contribution to the field, I would suggest the authors re-evaluate if there are more analyses / results that they could provide the readers with, or if at least the data could be shared openly so that others could support in exploring this valuable data set.

Thank you for the thoughtful feedback and acknowledgement of the value of the dataset and the potential contribution of this study to the field. We appreciate your recommendations and have conducted the requested analyses.

Reviewer #3 (Remarks to the Author):

This study reports on the impact of a maternal prenatal depression intervention (brief interpersonal psychotherapy) on infant amygdala and hippocampus volumes. The research question investigated is important, and findings have important implications for preventative interventions for children's health and wellbeing. The manuscript was also well written. I reviewed this work together with one of my doctoral students, and our amalgamated comments are below.

Major points
1. It is a hypothesis that reduced depressive symptoms led to hippocampal volume change. However, currently, this is not tested. Were hippocampal volumes correlated with depressive symptom change? Were there other symptoms (e.g., anxiety symptoms) or measures of stress assessed before and after the intervention?

We have implemented the reviewer's important suggestion to make our language precise. We now clarify throughout the manuscript that we test the effect of the prenatal intervention on hippocampal and amygdala volume. See example below:

Pg. 3. "The current innovative study employed a randomized clinical trial (RCT) to rigorously test the fetal origins hypothesis, aiming to determine whether an effective psychotherapy intervention for depression during pregnancy can yield significant effects on child neurodevelopment [3,3]. This research provides the first experimental evidence that intervention during pregnancy influences early offspring brain structure, highlighting the intergenerational impact of prenatal mental health treatment."

Further, we appreciate the reviewer's suggestion to add new analyses to directly test whether changes in depressive symptoms were associated with hippocampal volume. We examined whether depressive symptom slopes across pregnancy, based on repeated assessments of the Edinburgh Postnatal Depression Scale (EPDS) and SCL-20, were associated with infant hippocampal volume and found no significant associations across amygdala and hippocampal volumes.

Additionally, as requested, we test other maternal mental health symptoms using the State-Trait Anxiety Inventory (STAI) and stress using the Perceived Stress Scale (PSS) across pregnancy. Similarly, associations between anxiety and perceived stress symptom trajectories and infant amygdala or hippocampal volume were not significant.

These results are now referenced in the main manuscript and full models are included in the Supplement, **Supplementary Tables 8.1-8.4.**

Pg. 12: “To test whether changes in depressive symptoms across pregnancy were associated with infant amygdala and hippocampal volume, symptom trajectories, assessed via the SCL-20 and EPDS, were entered as separate predictors in the model. In addition to depression, other mental health indicators, including trajectories of maternal anxiety (assessed via the STAI) and stress (assessed via the PSS), were also examined as model predictors.”

Pgs. 13-14. “Depressive symptom slopes across pregnancy, based on repeated SCL-20 and EPDS assessments, were not significantly associated with infant hippocampal or amygdala volume. Similarly, there were no significant associations between STAI and PSS slope across pregnancy and infant hippocampal or amygdala volume (see **Supplementary Tables 8.1-8.4** for full model results).

We also now clarify in the discussion section that the tested pathways, namely prenatal maternal depression, anxiety, and perceived stress did not mediate the observed group differences in amygdala or hippocampal volume. We note that alternative mechanisms, such as maternal hormones or sleep during pregnancy, warrant further investigation. We now clarify that our manuscript identifies group differences in neonatal brain volume related to randomization to a prenatal treatment vs a control group but that the mechanism underlying this effect is not known.

Pg. 16-17, “The present findings underscore the importance of the intrauterine environment and the intergenerational impact of intervening during pregnancy on offspring brain development. We find prenatal group assignment to treatment vs. enhanced usual care predicted hippocampal volume. It is plausible that experiences in the early postpartum period related to treatment group contributed to the observed associations. Although the effect of IPT on maternal depression reduction may have persisted to the MRI assessment at 6 weeks postpartum, postnatal maternal depression symptoms did not predict infant hippocampal or amygdala volume. Further, covarying postnatal maternal depression did not alter the pattern of findings. Notably, the pathways tested within the current study, namely symptoms of depression and anxiety as well as perceived stress, were not significantly associated with infants’ amygdala or hippocampal volume. It may be surprising that maternal prenatal depression trajectories were not associated with amygdala or hippocampal volume. It is possible that the current study was underpowered to detect such effects. Alternatively, it is plausible that in addition to the effects of treatment on depression, the psychosocial intervention altered pregnant individuals’ biological processes (e.g., HPA axis signaling, inflammatory processes) and/or health behaviors (e.g., sleep, diet, and physical activity) that then impacted fetal development. Disruption to the maternal-placental-fetal HPA axis during gestation alters production of stress-responsive hormones, including cortisol and placental corticotropin-releasing hormone (for review see [77]). As these hormones cross the blood-brain barrier, they influence many processes critical to fetal brain maturation, including neuronal dendritic growth, myelination, and neurogenesis [78,79]. Depression is also associated with increased inflammation and altered cytokine levels[80–82]. which have been linked to child mental health [83–85] and brain development [86]. Maternal sleep, diet, and physical activity during pregnancy have also been linked to both maternal prenatal depression[87] and infant brain and behavioral outcomes [41,66,88–90], and may therefore represent alternative contributors to intervention effects.”

2. While scans were performed soon after birth (~6 weeks), it is still possible that the early postnatal environment could contribute. Could the authors examine the effect of IPT on postnatal maternal depression and the effect of postnatal maternal depression on infant brain structure? This would help to better disentangle the effects of prenatal vs postnatal maternal

psychopathology on infant outcomes and biological vs environmental influences on brain development.

We appreciate the reviewers' suggestion and have investigated the role of postnatal maternal psychopathology by examining 1) the effect of IPT on postnatal maternal depression at the time of scan, and 2) adding maternal postnatal depression as a covariate to the model.

Analyses demonstrated an effect of IPT on postnatal depression assessed at 6 months via the EPDS ($F(1,106) = 3.64$, $p = 0.059$; IPT: M =6.88, SE = 0.0.72, EUC (M =8.79, SE = 0.69). When we added postpartum depression to the model, we found that it did not predict right hippocampal volume ($F(1,102) = 0.00$, $p = .9678$) and treatment group remained a significant predictor ($F(1,102) = 9.32$, $p = .0029$).

We now include postnatal EPDS at 6 months as an additional covariate to the model (see **Supplementary Table 6** for full model results and reference these findings within the results and discussion:

Pg. 11-12, "Sensitivity analyses demonstrated that the intervention effects on hippocampal and amygdala volume remained consistent with the original model after additionally covarying for …. Additionally, models were also robust when covarying for postnatal EPDS (**Supplementary Table 6)."**

Pg. 16, "Although the effect of IPT on maternal depression reduction may have persisted to the MRI assessment at 6 weeks postpartum, postnatal maternal depression symptoms did not predict infant hippocampal or amygdala volume. Further, covarying postnatal maternal depression did not alter the pattern of findings."

3. In the analysis, covariates included postconception age at scan, intracranial volume, infant sex, and parity. I wonder if the authors have considered other confounding factors, such as maternal physical health, obstetric complications, and infant birthweight.

We thank the reviewer for this suggestion. As suggested, we have now also included the following additional covariates to our main group analyses: maternal physical health as indicated by maternal pre-pregnancy body mass index (BMI); diagnosis of maternal diabetes or hypertensive disorders (binary yes/no); obstetric complications (e.g. infection and placental abnormalities) and infant birthweight. These covariates were tested in addition to the originally included covariates which were postconceptional age at scan, ICV, infant sex, and parity. Results were consistent with the inclusion of these additional covariates.

We reference this sensitivity analysis within the main manuscript and include the full models within the supplement.

Pg. 11-12. "In sensitivity analyses, other potential confounding factors, including maternal physical health (i.e., diagnosis of hypertension and diabetes), body mass index (BMI), obstetric complications, infant birthweight ….were included as additional covariates."

Pg. 13. "Sensitivity analyses demonstrated that the intervention effects on hippocampal and amygdala volume remained consistent with the original model after additionally for … maternal health, BMI, obstetric complications, and infant birthweight (**Supplementary Table 4**).

Further, were QC scores correlated with subcortical volumes? The authors may wish to include these scores as covariates to increase specificity in the outcome variable.

We thank the reviewer for raising the issue of image quality. Imaging data were processed under the supervision of co-author Dr. Martin Styner, a leading expert in neonatal neuroimaging, using rigorous, standardized procedures. Image quality control was assessed with an in-house 4-point scale tailored for infant scans. Scans rated 0 (indicating excessive motion) were excluded (n=3). Images underwent standard preprocessing, including bias correction, atlas registration, and multimodal segmentation. Brain masks were manually reviewed and corrected. No scans were excluded due to failed segmentation of the hippocampus or amygdala. These procedures align with current best practices and ensure high-quality data.

In sensitivity analyses, we have now included image quality as a covariate in our primary models. Previously, scans with a score of 0 were removed. We now additionally include QC scores (1–3) as a covariate in all relevant models to account for residual variation in image quality. Image quality was not significantly associated with amygdala or hippocampal volume and all intervention effects remained consistent with original models (see **Supplementary Table 7**).

Related, were there any multicollinearity issues with both sex and intracranial volume being included in the model together?

Sex and ICV were modestly correlated ($r = 0.29$, $p = .002$). To assess multicollinearity, we calculated the condition index. The condition index for a model including only sex and ICV was 18.27, and for the full model including all covariates, the condition index was 25.38. Both values (i.e., 18.27 and 25.38) fall below the commonly accepted threshold of 30, indicating weak dependencies among predictors and no evidence of problematic multicollinearity (Kim et al. 2019 and Liao, 2012). Therefore, both sex and ICV were retained in the model, as there were no issues with multicollinearity.

4. Given that prior research has found sex differences in the association between maternal prenatal depression and subcortical volumes, did the authors consider looking at sex differences in their own analyses?

We appreciate this suggestion also requested by Reviewer 2. We now include analyses testing the interaction with sex. Sex did not significantly moderate the effect of treatment on hippocampal or amygdala volume. For the full models including tests of simple slopes, please see our response to Reviewer 2, comment #2 and the supplement.

5. Please provide a table outlining statistics for four full models, including the effects of covariates. It would be of interest to know if intracranial volume differed between groups.

Please see **Supplementary Table 2** for a table outlining statistics for the four full models, including the effects of covariates.

There was no significant difference in intracranial volume between groups $t(107) = 0.02$, $p = .987$, 95% CI [–27,101.8, 27,571.2], $d = 0.003$. We have added this information to the results section, see page 13.

Pg. 13. “There was no significant difference in intracranial volume between IPT (M = 552,262 mm³, SD = 80,010) and EUC (M = 552,027 mm³, SD = 63,639), $t(107) = 0.02$, $p = .987$, 95% CI [–27,101.8, 27,571.2], $d = 0.003$.”

6. Were there any statistical outliers, and were residuals from models normally distributed?

There were two values that were ±3 SD for the right hippocampus and one value for the left hippocampus. No outliers were found for the amygdala. Sensitivity analyses showed comparable results when these outliers were removed showing a significant treatment effect in the right hippocampus (right hippocampus: $t(101) = -2.97$, $p = 0.0038$) and no effect in the Left hippocampus: $t(102) = 1.34$, $p = 0.250$).

We include additional models with outliers removed in the supplement and reference these in the main text.

Pg. 11. “Exclusion of two values that were ±3 standard deviations for the right hippocampus and one value for the left hippocampus did not alter the effect size or significance of these analyses (see **Supplementary Table 3** for details).”

We conducted Shapiro-Wilk tests to assess the normality of residuals for each of the subcortical volume models. All p-values exceeded the conventional threshold of 0.05, indicating no significant departures from normality.

We include tests of normality in the methods section on page 13.

Pg 13. “Model-based residuals indicated no deviation from multivariate normality, as assessed by the Shapiro-Wilk test[67]: (SW range = 0.967-0.988, p value range = 0.1-0.49).”

7. Are the results adjusted for multiple comparisons? If not, please provide justification.

While our analyses initially focused on a small number of a priori, theory-driven regions of interest (bilateral amygdala and hippocampus), we have now applied a stringent Bonferroni correction to adjust for multiple comparisons across the four ROIs. This yields a corrected significance threshold of $\alpha = 0.0125$. Notably, the group difference in right hippocampal volume remains significant under this more stringent threshold.

We have added the following to the Methods section:

Pg. 11 “To account for multiple comparisons across the four subcortical regions (bilateral hippocampus and amygdala), a Bonferroni correction was applied (corrected $\alpha = .0125$).

8. Have the authors collected any infant behavioral and socioemotional data? It would be interesting to examine whether brain structure may mediate the effect of IPT intervention on infant outcomes.

We thank the reviewer for this suggestion. In response, we now include secondary analyses to examine infant negative emotionality at 6 months as a socioemotional outcome. Infant negative emotionality was assessed via standardized laboratory-based observations. Infants completed a validated task, “the Robot task” from the Laboratory Temperament Assessment Battery (Lab-Tab) designed to assess individual differences in negative emotionality and their behavioral responses were scored. We tested whether right hippocampal volume, which was significantly lower in the IPT group, was associated with negative emotionality. Results show that lower right hippocampal volume was significantly related to higher negative emotionality at 6 months (r=.19, p=.045). This finding extends our primary results and suggests that structural brain differences may have early behavioral consequences. These results have been added to the revised manuscript.

Pgs. 8-9, “Behavioral assessment of infant negative emotionality was assessed using the Robot Behavioral Task from the Laboratory Temperament Assessment Battery (Lab-TAB) [55]. The Robot Behavioral Task is a standardized and validated measure involving exposure to a robot that moves and makes noises. Infant responses to the robot were video-recorded and subsequently coded for intensity of facial negative affectivity (0–4) and distress vocalizations (0–5). Scores across facial and vocalization intensity were standardized and averaged to create a composite score of infant negative emotionality, with higher scores indicating higher negative emotionality. Twenty percent of recordings were double-coded by independent raters, with excellent interrater reliability (.94).”

Pg. 12. “Secondary analyses examined whether …3) neonatal amygdala and hippocampal volume were associated with infant negative emotionality at 6 months. ….Finally, partial correlations adjusting for intracranial volume and postconceptional age at scan were conducted to examine associations between subcortical volume and infants’ observed negative emotionality, assessed via laboratory-based observation at 6 months.

Pg. 14. “Partial correlations, conducted with left and right amygdala and hippocampal volumes and infant negative emotionality, showed that right hippocampal volume (r = .19, p = .045) and left hippocampal volume (r = .32, p < .001) were positively associated with negative emotionality. No significant associations were found for right amygdala volume (r = .08, p = .41) or left amygdala volume (r = –.002, p = .99).”

Pg. 15. “To test the functional correlates of enlarged hippocampal volume, we examined links with negative emotionality assessed via behavioral observation at 6 months of age. Findings showed that larger neonatal hippocampal volume was associated with greater subsequent infant negative emotionality. These preliminary data provide evidence that larger hippocampal volume may have implications for emerging emotional development. However, the long-term developmental significance and biological mechanisms underlying these differences are untested.”

Minor points
1. Incorrect in-text referencing on page 1.

The reference has now been fixed.

2. Measures: (1) It would be helpful to include internal consistency; (2) the time points when the measures were administered are not clear.

We have now included measures of internal consistency for EPDS and SCL-20.

Pg. 8, “Internal consistency for the EPDS was greater than .8 across all 5 timepoints. SCL-20: Pg 8, “Internal consistency was excellent (α > .9 across all three timepoints).”

Additionally, we have now added the weeks’ GA when the depression assessments were administered and provided an additional Supplementary Figure to illustrate a timeline of study data collection.

Pg. 7, “Participants completed self-report depression measures via REDCap three to five times across pregnancy: 16.41 (4.08) weeks (prior to intervention), followed by subsequent assessments at: 22.10 (4.01) weeks, 25.61 (4.08) weeks, 29.42 (4.18) weeks (near the end of active IPT), and 35.73 (1.58) weeks (end of gestation). The EPDS was administered at 6 weeks postpartum, concurrent with the MRI visit; see **Figure 2**. “

3. Does Figure 3 depict raw hippocampal volumes or volumes with covariates regressed out?

We appreciate the opportunity to clarify that Figure 3 originally depicted raw hippocampal volumes; however, we have now updated the figure to depict volumes with residuals regressed out and updated the figure legend to denote the plot with residuals regressed out. The figure depicting raw regional volumes has now been moved to the Supplement (**Supplementary Figure 2**).

4. On page 4, the authors state “While such accelerated maturation …these changes may increase the lifetime risk of mental illness and have been associated with behavioral outcomes such as increased negative emotionality and cognitive impairments 22,23” These studies looked at white matter and not subcortical volume, so relevance is unclear.

We thank the reviewer for pointing this out. Given the limited infant neuroimaging literature, few studies have directly linked prenatal depression-related brain changes to infant behavioral outcomes. We have now added another citation specific to volumetric alterations to improve clarity and relevance (Miller et al. 2024).

Pg. 4, “While accelerated maturation may initially be adaptive in meeting the demands of the current environment [40], neurodevelopmental changes may increase the lifetime risk of mental illness and, in fact, have been associated with socioemotional outcomes such as increased negative emotionality and depressive symptoms [41,42].”

REVIEWER COMMENTS

Reviewer #1 (Remarks to the Author):

The authors very effectively responded to the reviewers' feedback, including the excellent comments made by other reviewers. This is a clearly-written paper reporting interesting, novel, and important results.
However, I am a bit troubled by the lack of association between change in maternal mood and infant hippocampal volume, esp in the context of 1 out of 4 a priori brain hypotheses being supported, albeit there now is correction for multiple comparisons.
In addition to the interpretations offered, perhaps the authors should consider: failure of randomization that is not detected with current subject phenotyping; the role of social support experienced via IPT (perhaps this even could be tested?).

We appreciate the reviewer's thoughtful comments and careful consideration of alternative hypotheses. To address the possibility that our findings may reflect undetected failures of randomization, particularly regarding race composition or poverty, we conducted additional analyses following the reviewer's suggestion. First, we confirm that there are no significant group differences that would suggest a failure of randomization on any of the sociodemographic variables tested. We had previously presented that including parity as a covariate does not affect study findings (See Supplementary Table 2). To probe the possibility that small, but non-significant group differences in race and ethnicity or poverty contributed to RCT findings, we conducted sensitivity analyses by including race and poverty (in addition to parity) as covariates in our primary models. In adjusted models, neither poverty ($F(1,101) = 1.10$, $p = 0.2966$), nor race ($F(1,101) = 0.02$, $p = 0.8976$) were significantly associated with hippocampal volume. Further, the statistical significance of the intervention effect was unchanged with these additions.

- Right hippocampal volume: $p = .0023 \rightarrow .0024$
- Left hippocampal volume: $p = .1613 \rightarrow .1696$
- Right amygdala volume: $p = .8344 \rightarrow .8307$
- Left amygdala volume: $p = .4867 \rightarrow .4713$

We sincerely appreciate the reviewer's thoughtful suggestion regarding the potential role of social support. The trial was explicitly designed and powered to test the effects of the intervention on prenatal depression as the core target of IPT. Accordingly, our mechanistic analyses focused on depression. In response to reviewer requests on our first submission, we added other validated indicators of maternal distress (anxiety and perceived stress). We clarify in the manuscript that these pathways did not account for the intervention effect. We agree that other plausible mechanisms including IPT-related improvements in social support remain a theoretically meaningful and plausible mechanism. Testing the broad range of plausible physiological and behavioral mediators is beyond the scope of this investigation. We have added discussion of the need for future work evaluating a broader set of mediators, including social support, to fully elucidate the pathways through which prenatal psychotherapy influences fetal neurodevelopment.

Pg. 16, "Notably, the pathways tested within the current study, namely symptoms of depression and anxiety as well as perceived stress, were not significantly associated with infants' amygdala or hippocampal volume. It may be surprising that maternal prenatal depression trajectories were not associated with amygdala or hippocampal volume. It is possible that the current study was underpowered to detect such effects. Alternatively, it is plausible that in addition to the effects of treatment on depression, the psychosocial intervention altered pregnant individuals' biological processes (e.g., HPA axis signaling, inflammatory processes), health behaviors (e.g., sleep, diet, and physical activity) and/or IPT-related skill development (e.g., communication and social support) that then impacted fetal development. In terms of biological pathways, disruption to the maternal-placental-fetal HPA axis during gestation alters production of stress-responsive hormones, including cortisol and placental corticotropin-releasing hormone (for review see [77]). As these hormones cross the blood-brain barrier, they influence many processes critical to fetal brain maturation, including neuronal dendritic growth, myelination, and neurogenesis [78,79]. Depression also is associated with increased inflammation and altered cytokine levels[80–82] , which have been linked to child mental health [83–85] and brain development [86]. It also is plausible that IPT alters maternal health behaviors, including sleep, diet, and physical activity[3,87]. Alteration of any of these processes would have downstream impacts on the developing

fetal brain and behavior[41,66,88,89]. Finally, IPT targets social skill development, and improvement of social skills can both increase social support [90] and decrease sources of stress related to interpersonal relationships[91,92]. The present study identifies important impacts of randomization on infant brain development. Future work is needed to determine the mechanism underlying the effect of IPT treatment on infant development."

Finally, I do have a question about the group comparison in Table 1 that maybe could account for the lack of effect of mood improvement in relation to infant MRI outcomes: the only statistically significant difference between groups is parity/being nulliparous; however, when you look at the % in the rows of demographic variables, there are hints of differences: nearly 2x the number of Hispanic/Latine in the EUC group; > Blacks and Asians in the EUC group; higher % White in the IPT group; and then > percent experiencing poverty in the IPT group (could this be triple checked? In the USA, as we all know, people of color tend to experience higher rates of poverty yet the IPT group with a higher % of White participants has a higher % of people experiencing poverty). Putting demographics and parity together, to the extent that having more children is stressful, and being a person of color in the USA is stressful, it would seem the EUC group is overall experiencing more stressors/life stress. Are these 'soft markers' of group differences that may not be affected by IPT yet relate to infant hippocampal volume? Independent of group assignment, are these variables related to infant hippocampal volume?

We can confirm that the numbers in Table 1 are correct and that there are not significant group differences that would support a failure of randomization. It is the case that the number of participants living below the poverty line and the percentage of white participants is higher (although not significantly) among the IPT group. We appreciate the broad array of social and contextual factors that impact both mental health and infant brain development. Although we are cautious about interpreting these non-significant group differences, we sought to rigorously probe the reviewer's important alternative hypothesis that cumulative social stressors account for study findings. First, as noted above, we included income-to-needs ratio, race, and parity (number of live births) in our analyses as covariates. Inclusion of these indicators of social stress did not alter findings related to the effects of IPT.

To specifically address the reviewer's comment *"Putting demographics and parity together, to the extent that <u>having more children is stressful</u>, <u>and being a person of color in the USA is stressful</u>, it would seem the EUC group is overall experiencing more stressors/life stress"*, we created a stressor index which consisted of a point scale: 0 = no stressor: first child and non-Hispanic White; 1 = any stressor: having children and/or identifying as a person of color or Hispanic. We examined the interaction between this stressor index and intervention assignment on right hippocampal volume. The interaction was non-significant ($F(1,102) = 0.03$, $p = .87$). Further, model-based estimates showed similar intervention effects within each group. Model-based estimates and contrasts within the non stressor group yielded hippocampal volume metrics of 1145.05 (se=24.57) for IPT and 1220.80 (se=43.07) for EUC with a difference of 75.74 (se=49.34, $t(102)=1.54$, p=0.13). Model-based estimates and contrasts within the stressor group yielded 1112.94 (se=17.94) for IPT and 1179.67 (se=14.65) with a difference of 66.73 (se=23.29, $t(102)=2.87$, $p=0.05$). These analyses indicate that the intervention effects were comparable between "stressor" groups.

We value these additional questions regarding randomization. As stated by the reviewer, it is known that sociocontextual factors affect mental health. We have added to the discussion the importance of considering depression treatment in the context of sociocontextual factors in future studies.

Pg. 18, "Finally, accumulating evidence suggests that sociocontextual factors, including experiences of poverty and broader structural stressors influence maternal mental health during pregnancy[95,96] and infant neurodevelopment [97,98]. The current intervention, MOMCare, was designed to meet the needs of pregnant individuals experiencing sociocontextual stressors [47]. Future research may investigate how contextual factors amplify or attenuate treatment effects and will help to tailor interventions for those facing the greatest adversity."

Reviewer #2 (Remarks to the Author):

The authors have addressed all my points.

Reviewer #3 (Remarks to the Author):

We thank the authors for their responses to our comments, and for their comprehensive revision. We believe the manuscript is acceptable for publication in its current form, and will make an excellent contribution to the literature.

Reviewer #4 (Remarks to the Author):

I co-reviewed this manuscript with one of the reviewers who provided the listed reports. This is part of the Nature Communications initiative to facilitate training in peer review and to provide appropriate recognition for Early Career Researchers who co-review manuscripts.
